# Diastereodivergent Catalysis

**DOI:** 10.1021/jacsau.3c00216

**Published:** 2023-09-26

**Authors:** Daniel Moser, Tanno A. Schmidt, Christof Sparr

**Affiliations:** Department of Chemistry, University of Basel, St. Johanns-Ring 19, 4056 Basel, Switzerland

**Keywords:** atropisomers, diastereomers, dual catalysis, higher-order stereogenicity, stereocenters, stereoselective catalysis

## Abstract

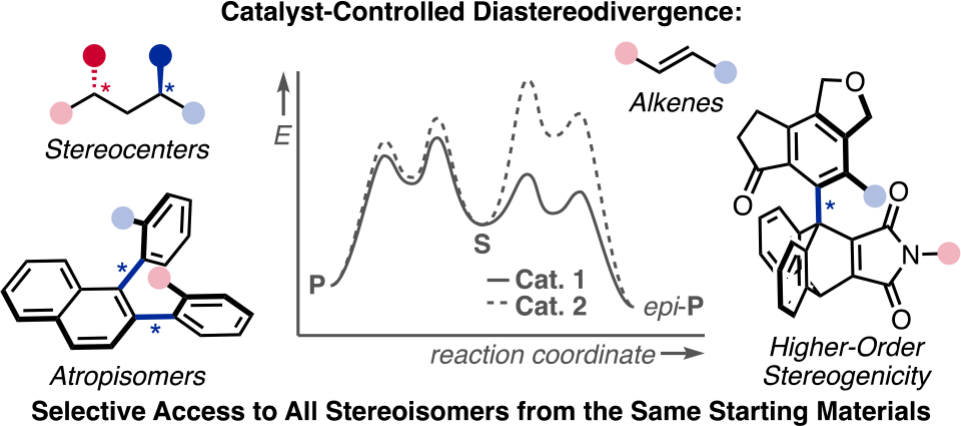

Alongside enantioselective catalysis, synthetic chemists are often confronted by the challenge of achieving catalyst control over the relative configuration to stereodivergently access desired diastereomers. Typically, these approaches iteratively or simultaneously control multiple stereogenic units for which dual catalytic methods comprising sequential, relay, and synergistic catalysis emerged as particularly efficient strategies. In this Perspective, the benefits and challenges of catalyst-controlled diastereodivergence in the construction of carbon stereocenters are discussed on the basis of illustrative examples. The concepts are then transferred to diastereodivergent catalysis for atropisomeric systems with twofold and higher-order stereogenicity as well as diastereodivergent catalyst control over *E*- and *Z*-configured alkenes.

## 1 Introduction

Introducing multiple stereogenic elements into molecular frameworks leads to an exponentially increasing number of stereoisomers, as expressed by the renowned Le Bel–van ‘t Hoff rule.^1,2^ Arrays of stereogenic units such as stereocenters or stereogenic axes are present in a multitude of natural products, bioactive compounds, and building blocks for nanomaterials. Therefore, chemists strive to find efficient ways to individually control the configuration of each stereogenic unit within a molecule and to access all possible stereoisomers, which likely possess different properties of interest. Fully stereodivergent methods are therefore necessarily required, and catalyst stereocontrol is considered as an ideal approach toward this aim. *Full stereodivergence* thereby describes the ability to control a stereoselective reaction in a way that any enantiomer or diastereomer can be obtained selectively as the major product from identical starting materials. This implies the requirement for complete *enantio*- and *diastereodivergence*, so that the absolute configurations of a product can be inverted, while in addition, diastereodivergence redirects the outcome between the crucially different diastereomeric products to define the relative configuration.

**Figure 1:**
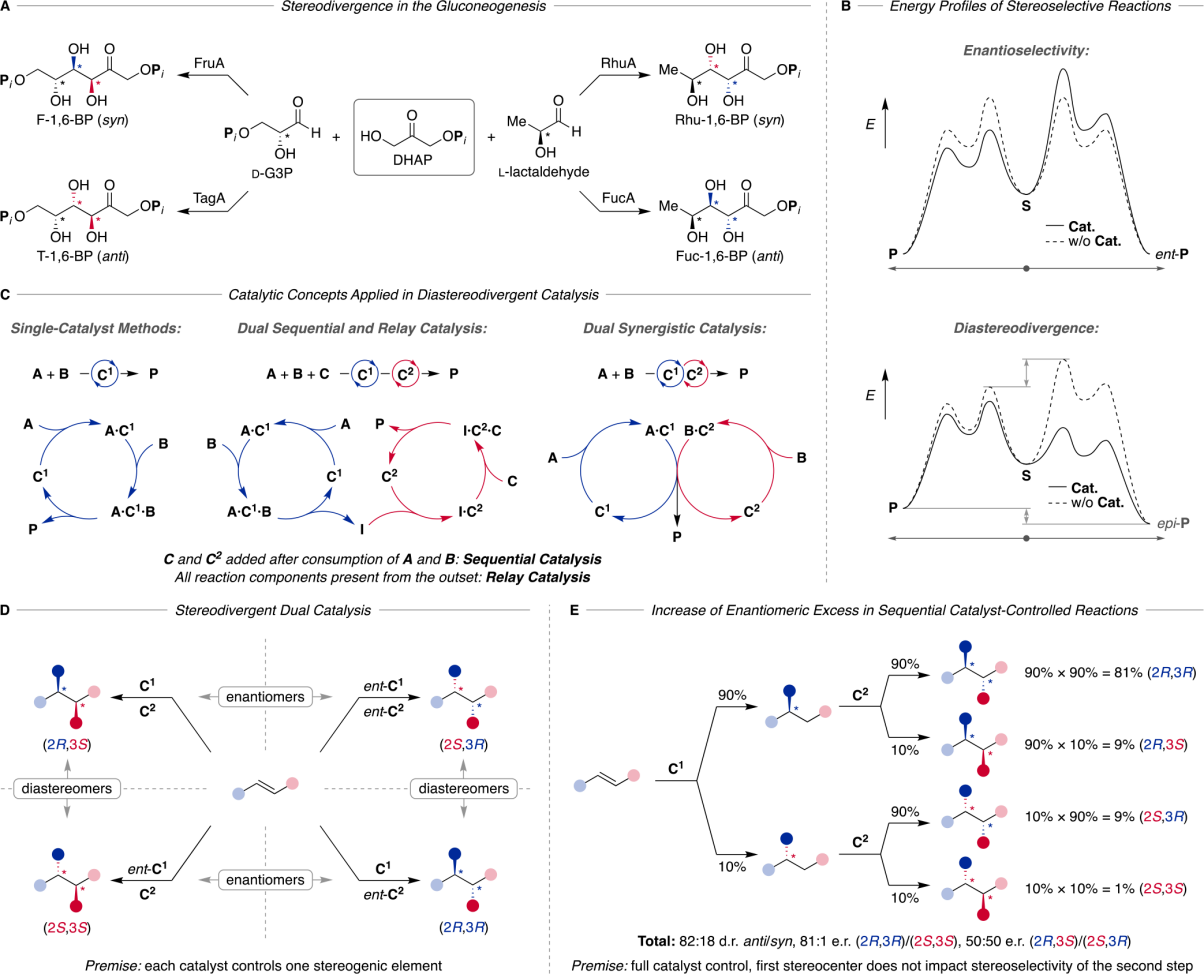
Key concepts in diastereodivergent catalysis: (A) enzyme catalyzed stereodivergent aldol reaction in gluconeogenesis, (B) schematic energy profiles of an enantioselective and a diastereodivergent reaction, (C) catalytic concepts applied in diastereodivergent catalysis, (D) permutations of catalyst configurations give rise to four stereoisomers in a diastereodivergent dual catalytic reaction, and (E) Horeau principle for the e.r. amplification in a sequence of two stereoselective reactions.

In natural biosynthetic pathways, enantio- and diastereoselectivities are typically controlled by sophisticated enzymatic reactions. A fascinating example for the aptitude of enzymatically controlled diastereodivergence is witnessed for gluconeogenesis, in which the configuration of two new stereocenters is governed in the aldol addition of dihydroxyacetone phosphate (DHAP) to d-glyceraldehyde 3-phosphate (d-G3P) or l-lactaldehyde (Figure 1A, (*S*): red, (*R*): blue).^3^ While d-fructose 1,6-bisphosphate aldolase (FruA) provides *syn*-configured d-fructose 1,6-bisphosphate from d-G3P, which is further transformed into d-glucose, d-tagatose 1,6-bisphosphate aldolase (TagA) converts the same substrate d-G3P diastereodivergently into the *anti*-configured product. In a similar fashion to FruA and TagA, however, starting from l-lactaldehyde, the enzymes RhuA and FucA convert DHAP diastereodivergently to rhamnulose-1,6-bisphosphate (Rhu-1,6-BP) and fuculose-1,6-bisphosphate (Fuc-1,6-BP) with inverted stereoselectivity for the two newly formed stereocenters. Emulating the concepts of enzymatic reactions by small-molecule catalysis therefore aims to mimic control over the substrate orientation and stabilization usually with inner sphere interactions. The scenarios for diastereodivergence span between the extremes of low substrate bias—for which methods developed for enantioselective catalysis are typically directly applicable—to the particularly challenging cases of a strong catalyst–substrate mismatch—in which the inherent substrate bias toward a preferred diastereomer must be efficiently overcome by means of a catalytic activation. Whereas the sense of selectivity in enantioselective reactions is inverted by employing the enantiomeric catalyst, if available, the stereochemical outcome of diastereoselective reactions is often strongly impacted in proximity of configurationally defined stereogenic units of a substrate or intermediate (Figure 1B).

Two general approaches emerged to achieve diastereodivergence by using small-molecule catalysts. With stepwise control over the stereogenic units, the configuration of one stereogenic element is controlled at a time, allowing the choice of catalysts for every step. Ideally, this can be repeated to build up recuring structural motifs in an iterated reaction sequence consisting of a stereoselective reaction and the transformation of its product into a suitable substrate for the next iteration.

While the stepwise strategy allows for a simplified screening process, it is also lengthy and can require several purification steps. Conversely, the construction of two or more stereogenic units in a single transformation constitutes a very efficient means to obtain stereochemically well-defined and highly functionalized products by rapidly increasing the stereochemical complexity. Following this approach, single-catalyst methods or dual catalytic systems^4−8^ are conceivable (Figure 1C). Whereas in the former, one catalyst activates and guides two reaction partners through a distinct reaction pathway that selectively affords one of the multiple stereoisomers, the stereochemical outcome of the reaction in dual catalytic systems is determined by the combination of two catalysts. In the ideal case, each catalyst independently controls the configuration of one stereogenic unit, so that by choosing different combinations of the catalyst enantiomers, diastereomeric products are obtained selectively (Figure 1D). On the other hand, several challenges arise in the design of dual catalyst systems. First, the envisaged catalysts and reagents need to be compatible, i.e., deactivation reactions such as ligand exchange, acid–base, or redox reactions between them must be avoided. Furthermore, depending on the mechanism, catalyst selectivity for a specific substrate or intermediate is crucial to avoiding unspecific activation and side reactions. Mechanistically, three general modes of operation are distinguished in dual catalysis (Figure 1C). In sequential and relay catalysis, there are two catalytic cycles, with the first cycle generating an intermediate that subsequently enters the second catalytic cycle in one pot without isolation. While accumulation of the intermediate is possible, this can allow for shorter synthetic routes and the involvement of reactive intermediates. In relay catalysis, all starting materials and catalysts are present in the reaction mixture from the outset of the reaction, requiring that all reaction partners and catalysts are compatible with each other and do not interfere; i.e., catalysts, starting materials, and intermediates show orthogonal reactivity profiles. However, if this is not given, a sequential dual catalysis approach allows the avoidance of certain incompatibilities by the addition of a reagent and the catalyst for the second step once the starting materials of the first step have been consumed. Conversely, in synergistic catalysis, two starting materials are activated by two distinct catalysts—usually as electrophile and nucleophile—which then react with each other. Over the last years, the requirements for the development of dual catalytic reactions were met by several metal–metal, metal–organo, and organo–organo dual catalytic systems, which were successfully applied for different bond formation processes.

An advantage of combining two stereoselective reactions as in sequential and relay dual catalysis is that according to the Horeau principle, an overall enantiomeric ratio (e.r.) which is greater than the enantioselectivities of the individual reaction steps can be obtained.^9,10^ This is shown in Figure 1E under the premise of the absence of substrate stereocontrol, e.g., in the construction of remote stereocenters. As the second step does not yield enantiomeric but diastereomeric products from one stereoisomer of the intermediate, the overall enantioselectivity is 81:1 e.r., whereas the individual steps show stereoselectivities of 90:10. However, the high enantioenrichment is achieved at the cost of diastereoselectivity (82:18 d.r.) and consequently yield.

From this general background, the present Perspective aims at providing an overview of the concepts and current challenges in the development of catalyst-controlled diastereodivergent reactions illustrated by selected examples without the intention of a comprehensive account of the field, which has been the subject of several review articles.^11−17^ Pseudo-diastereodivergent reactions, in which substrates are changed to alter the stereochemical outcome, e.g., by changing alkene configuration,^18−22^ will therefore not be covered.

## 2 Iterative Catalyst Control over Multiple Stereocenters

Repeating structural motifs containing stereocenters are found in a variety of polyketide natural products.^23^ Since some of these natural deoxypolypropionate and polypropionate products are known to possess important biological activities, their stereocontrolled synthesis has received notable interest. As in their biosynthesis, an iterative approach toward their stereodivergent preparation has proven to be highly efficient. Chiral reagent-based iterative processes were established as reliable means to diastereodivergently access carbon chains with multiple stereocenters.^24−26^ Combined approaches^20^ and catalytic methodologies on the other hand are less developed, underlining the persisting challenges associated with catalyst diastereodivergence.

**Scheme 1:**
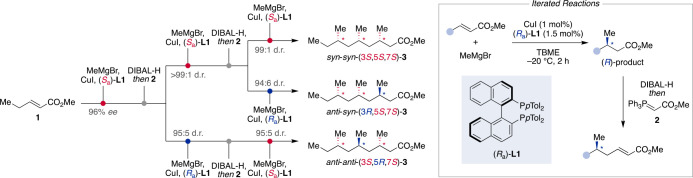
Iterative Diastereodivergent Control over Deoxypolypropionate Chains by a Conjugate Addition–Chain Prolongation Sequence

For instance, based on previous work by the Feringa group,^27^ Loh and co-workers found a simple and elegant way to build up polydeoxypropionate chains starting from acrylic ester **1** (Scheme 1).^28^ The iterative strategy comprises two steps, the stereoselective conjugate addition of MeMgBr to an α,β-unsaturated ester under catalyst control^29^ and subsequent chain prolongation to regenerate the α,β-unsaturated ester motif setting the stage for the next iteration of the reaction sequence. After a highly enantioselective Cu^I^/(*S*_a_)-**L1** catalyzed conjugate addition of MeMgBr to **1** (96% *ee*) and chain prolongation by a sequential one-pot ester reduction–Wittig olefination with **2**, the diastereoselectivity was diverged to yield the *syn*- or *anti*-products, respectively. While the reaction with (*S*_a_)-**L1** proceeded under highest diastereocontrol with a d.r. of >99:1 for the *syn*-product, the *anti*-product was obtained using the enantiomeric (*R*_a_)-**L1**. Representing the catalyst–substrate mismatch scenario, this reaction proceeded with only slightly diminished diastereocontrol (95:5 d.r.).^28^ Both diastereomers were extended to prepare for the introduction of the third stereocenter. Starting from the *syn*-(3*S*,5*S*)-product, the diastereoselectivity was diverged between *syn*-*syn*-**3** and *syn*-*anti*-**3** using the same reaction conditions as before and the products were obtained in similar d.r.’s (99:1 d.r. for *syn*-*syn*-(3*S*,5*S*,7*S*)-**3** with (*S*_a_)-**L1** and 94:6 d.r. for *anti*-*syn*-(3*R*,5*S*,7*S*)-**3** with (*R*_a_)-**L1**). Also, the *anti*-*anti*-diastereomer of **3** was obtained from the *anti*-deoxydipropionate intermediate in 95:5 d.r. with (*S*_a_)-**L1**. Notably, the diastereoselectivity of this step was comparable with the formation of *anti*-*syn*-**3**, and all stereoselective conjugate addition reactions–even of the extended substrates–were carried out with high diastereoselectivity under identical conditions.

**Scheme 2:**
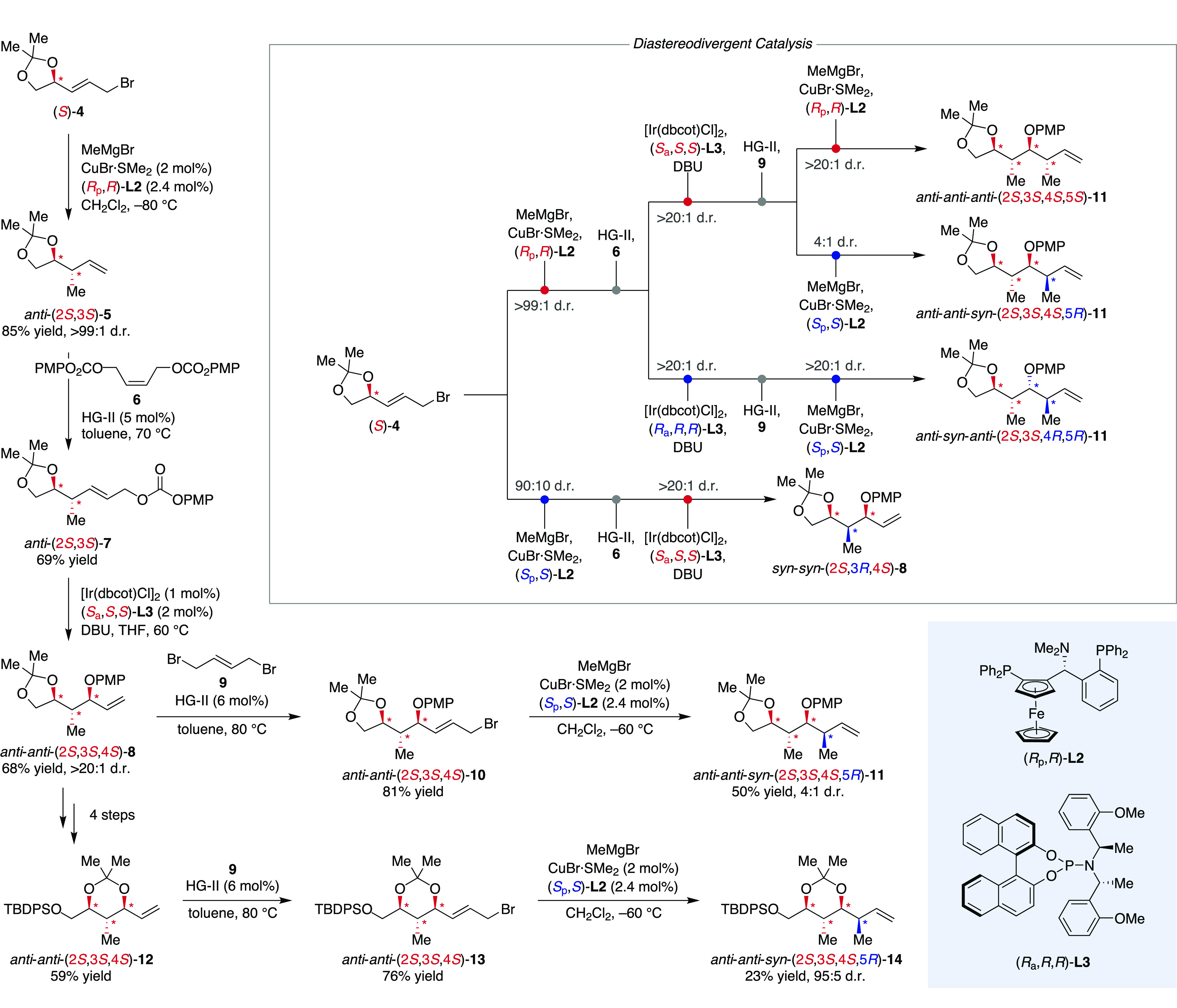
Diastereodivergent Access to Polypropionate Chains by Stepwise Construction of Vicinal Stereocenters dbcot = dibenzo[*a*,*e*]cyclooctene; HG-II = Hoveyda–Grubbs catalyst II; PMP = *p*-methoxyphenyl.

In oxygenated polypropionate chains, the 1,3-dimethyl substitution pattern is combined with vicinal hydroxy functionalities, yielding an array of 1-hydroxy-2-methylethylene units with all-vicinal stereocenters. For these, the Feringa group developed an iterative approach allowing for the alternating installation of vicinal methyl and hydroxy groups under catalyst control (Scheme 2).^30^ (*S*)-Configured allyl bromide **4** bearing an acetal protected glycol motif was subjected to Cu-catalyzed stereoselective allylic alkylation.^31,32^ In both the substrate–catalyst match and mismatch case, Taniaphos **L2** exerted high degrees of diastereocontrol and combination of (*S*)-**4** with Cu^I^/(*R*_p_,*R*)-**L2** yielded the matched *anti*-product (2*S*,3*S*)-**5** with perfect diastereoselectivity (>99:1 d.r.). In contrast, pairing with (*S*_p_,*S*)-**L2** afforded *syn*-**5** in an only slightly reduced d.r. of 90:10. The levels of stereocontrol achieved in the construction of the second vicinal stereocenter show the overall high degree of catalyst control. Beside very good diastereoselectivity, excellent regioselectivity for the linear product was observed, which however is largely governed by the substrate, as the comparison with other allylic bromides revealed.^32^ To prepare for the ensuing introduction of the next vicinal stereocenter, both diastereomers were subjected separately to cross-alkene metathesis with alkene **6**. The obtained allyl *p*-methoxyphenyl (PMP) carbonates **7** were then carried on to a decarboxylative stereoselective allylation^33,34^ under release of *p*-methoxyphenol as internal nucleophile. The Ir-catalyzed intramolecular decarboxylation–etherification afforded PMP-ether **7** carrying three contiguous stereocenters. Employing (*S*_a_,*S*,*S*)- and (*R*_a_,*R*,*R*)-**L3**, respectively, remarkable d.r.’s of >20:1 were obtained for both diastereomers from *anti*-(2*S*,3*S*)-**7**, and also the reaction of diastereomeric *syn*-(2*S*,3*R*)-**7** in the presence of (*S*_a_,*S*,*S*)-**L3** afforded a similar d.r. In addition to good diastereoselectivities, all reactions showed excellent regioselectivity for the linear product. Beginning the next iteration of the reaction sequence, the *anti-anti-* and *anti-syn-*diastereomers of **8** were expanded with **9** to allylic bromides **10** and resubjected to the Cu^I^/**L2**-catalyzed allylic substitution with MeMgBr. While the *anti*-*anti*-*anti*- and *anti*-*syn*-*anti*-configured products **11** were obtained in excellent diastereoselectivities with d.r.’s > 20:1, the reaction of *anti*-*anti*-(2*S*,3*S*,4*S*)-**10** with (*S*_p_,*S*)-**L2** showed distinct mismatch behavior and *anti*-*anti*-*syn*-(2*S*,3*S*,4*S*,5*R*)-**11** was obtained in a significantly reduced 4:1 d.r. However, the substrate bias could be circumvented by altering the protecting group strategy and six-membered cyclic acetal *anti*-*anti*-(2*S*,3*S*,4*S*)-**12** was obtained in four steps from *anti*-*anti*-(2*S*,3*S*,4*S*)-**8** allowing for pseudo-diastereodivergent access to *anti*-*anti*-*syn*-configured **14**.

## 3 Single-Catalyst Methods for the Simultaneous Control over Two Stereocenters

**Scheme 3:**
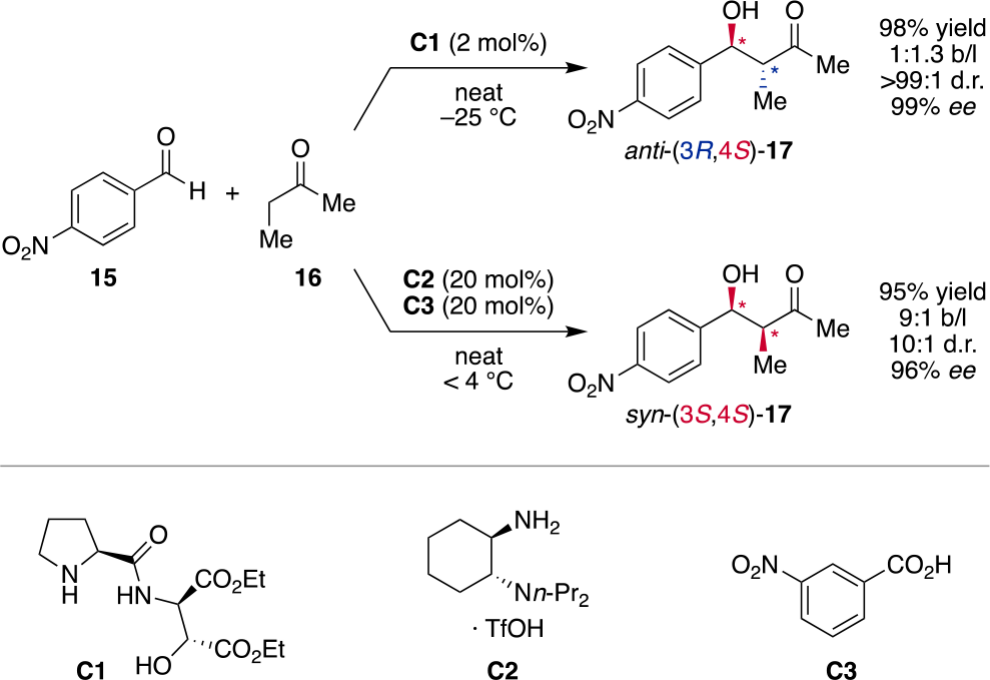
Diastereodivergent Aldol Addition Using One Chiral Catalyst for Simultaneous Control over Two Stereocenters

While the stepwise iterative control over stereocenters is suitable for repeating units with alternating configurations, manifold reactions in which two or more stereocenters are formed are well-known. Controlling diastereodivergence in these would be an efficient way to rapidly increase stereochemical complexity within a single step. Prototypically, the aldol reaction generates up to two new carbon stereocenters, giving rise to four possible stereoisomers, and catalyst control over their configuration has been a long-standing challenge. However, a unified approach toward full stereodivergence for direct aldol reactions has not emerged to date, but examples for divergent diastereocontrol were reported. An illustrative example for the challenges associated with the aldol addition is the reaction between 4-nitrobenzaldehyde (**15**) and butanone (**16**) where not only chemoselectivity between the linear and branched products resulting from nucleophilic activation of either of the α-positions of **16** needs to be controlled, but also the differentiation between the four stereoisomers of the branched product **17** is required (Scheme 3). Interestingly, the use of small bifunctional chiral amine catalysts such as **C1**,^35^ which activate the ketone as enamine and guide it to the aldehyde via a hydrogen-bond network, provided high *ee*’s of up to 99% and diastereoselectivities of >9:1.^36^

Single catalyst systems often encounter a challenge in stereoselective reactions that afford more than one stereogenic unit—the capability to form only one diastereomer while maintaining high enantioselectivity. Notably, the spectrum of potential reactions spans from 1,4-, 1,6-conjugate additions,^37^ aldol,^38,39^ to Mannich reactions and related transformations.^12^

**Scheme 4:**
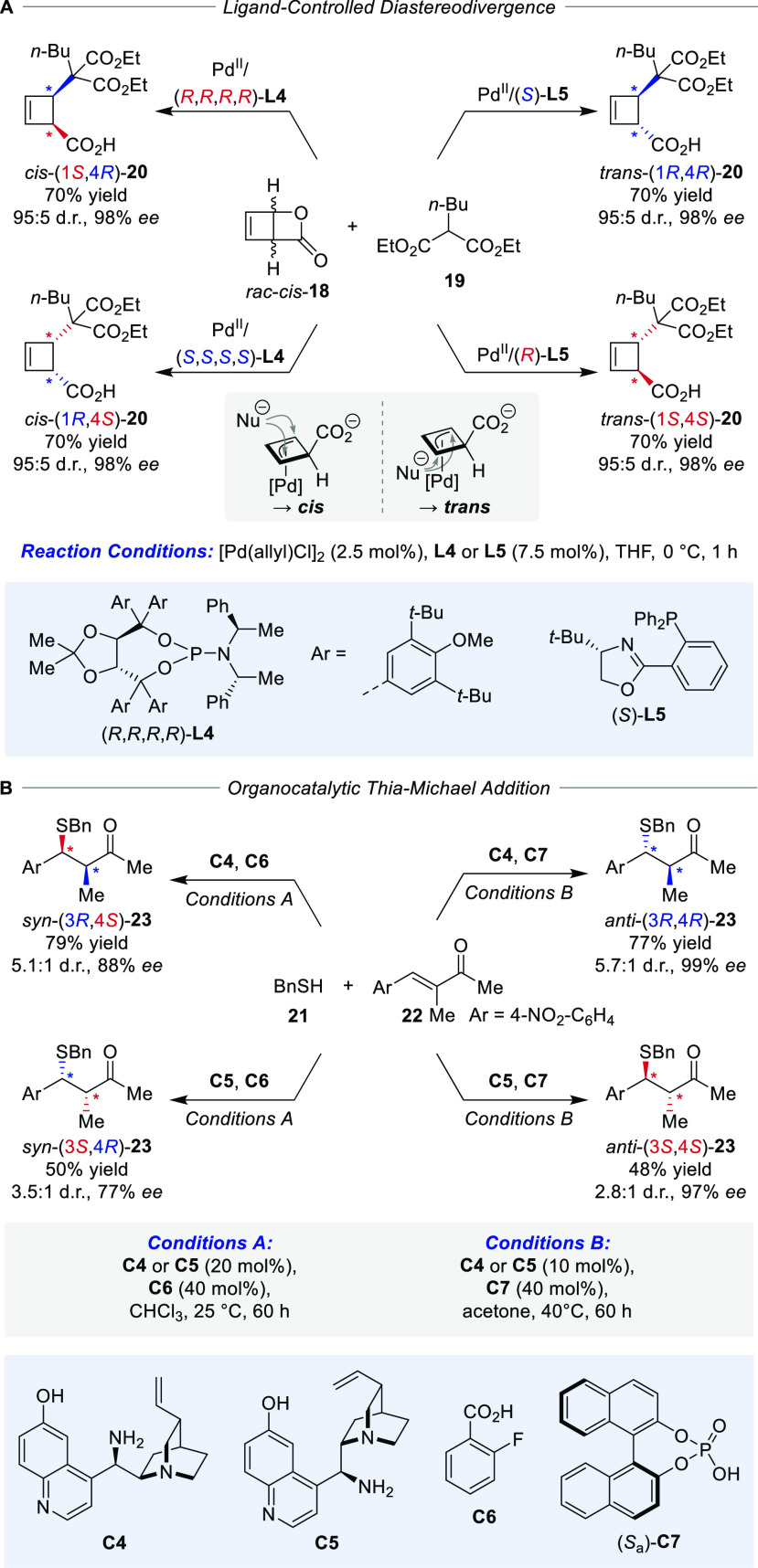
Representative Examples for Diastereodivergent Catalysis (A) Pd-catalyzed access to all cyclobutene stereoisomers from racemic starting material and (B) organocatalytic diastereodivergent thia-Michael addition.

Addressing the efficient control of two vicinal stereocenters by means of a single catalyst was realized by Maulide and co-workers in the diastereodivergent dynamic kinetic stereoselective transformation of strained *cis*-2-oxabicyclo[2.2.0]hex-5-en-3-one (**18**) by a Pd-catalyzed nucleophilic ring-opening with malonate **19** (Scheme 4A).^40^ Here, careful selection of ligands was the key to achieving diastereodivergence. While the TADDOL-based phosphoramidite ligand **L4** proved to be highly *cis*-selective, the structurally distinct PHOX ligand **L5** favored the formation of the *trans*-product. This illustrates the challenges of diastereodivergence using only a single catalyst, namely, that different mechanistic pathways are required, as reflected by the substantially different ligand scaffolds. Common for both the *cis*- and *trans*-selective reaction, the β-lactone is opened by the Pd-catalyst to form regioisomeric η^1^-allyl complexes which interconvert through the η^3^-isomer. This fluxional behavior results in the time-averaged planarization and symmetrization of the substrate, allowing access to all four stereocenter configurations. In the first report, the *cis*-configured products were assumed to arise via an overall stereoretentive conventional Tsuji–Trost mechanism by the formation of the allyl complex on the opposite face of the leaving group and subsequent S_N_2-like attack by the nucleophile on one of the enantiotopic termini of the allyl system, whereas the *trans*-selective mechanism would proceed through a stereoanomalous *syn*-addition of the Pd-catalyst to **18**. The mechanistic picture was more recently refined to exclusively involve Pd/carboxylate-*anti*-configured allyl complexes as catalytic intermediates, and the diastereoselectivity is determined by the ligands governing the face of nucleophilic attack on the allyl complex. The scope was expanded to more stable chlorocyclobut-2-ene carboxylic acid starting materials from which the same Pd-allyl intermediates as with **18** were formed independently of the absolute and relative configuration of the starting material.^44^ Even a mixture of the four possible starting material isomers could be selectively transformed into the four stereoisomers of **20**, underlining the stereoconvergent nature of the method.

A conceptually different approach for altering the selectivity to gain diastereodivergence was disclosed in a study by Melchiorre and co-workers for a stereoselective thia-Michael addition to α-branched enones, where efficient control over diastereoselectivity was achieved by applying an external chemical stimulus (Scheme 4B).^41^ Here, a suitable combination of acid cocatalyst and reaction medium was crucial for effectively changing the amine catalyst’s diastereoselection between the *syn*- and *anti*-product upon the formation of chiral ion pairs with distinct structural features depending on the nature of the acid. Based on the well-known property of the catalytic performance of cinchona scaffolds to show distinct behaviors depending on their conformation,^42^ it is rationalized that the two distinct acid cocatalyst **C6** and **C7** are able to induce conformational changes in the three-dimensional structure of the catalyst in solution, a phenomenon also known to occur with altered polarity of the reaction medium.^43^

**Scheme 5:**
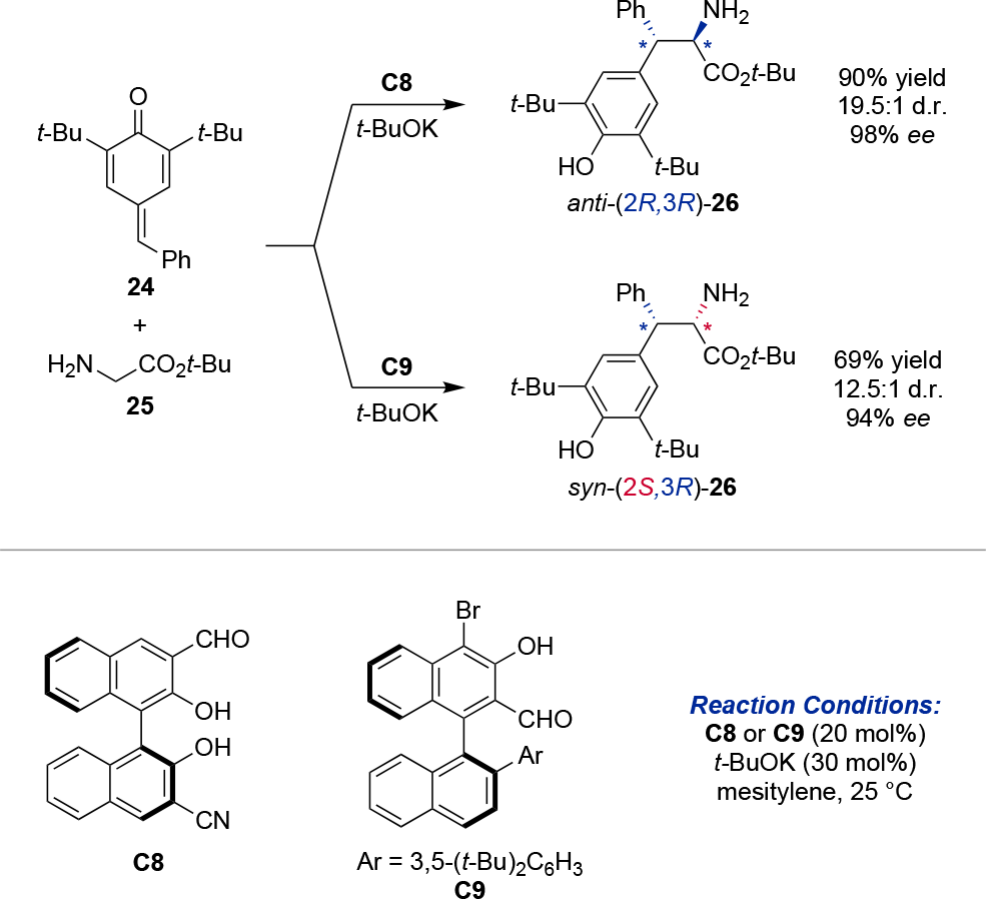
Diastereodivergent Synthesis of Nonproteogenic α-Amino Acids under Aldehyde Catalysis

An example for the potential of rationally designed single catalyst systems was disclosed recently for the diastereodivergent 1,6-conjugate addition of amino acids **25** to *p*-quinone methides (PQM) **24** and the related Mannich reaction of pyridinylmethamines and aldimines by the means of chiral aldehyde catalysis (Scheme 5).^37^ Density functional theory (DFT) guided catalyst design revealed 3-formyl BINOL aldehyde **C8** as an efficient catalyst for the *anti*-1,6-conjugate addition and *syn*-Mannich reaction. In contrast, 2-formyl binaphthalene catalyst **C9** showed an inverted diastereoselectivity.

**Scheme 6:**
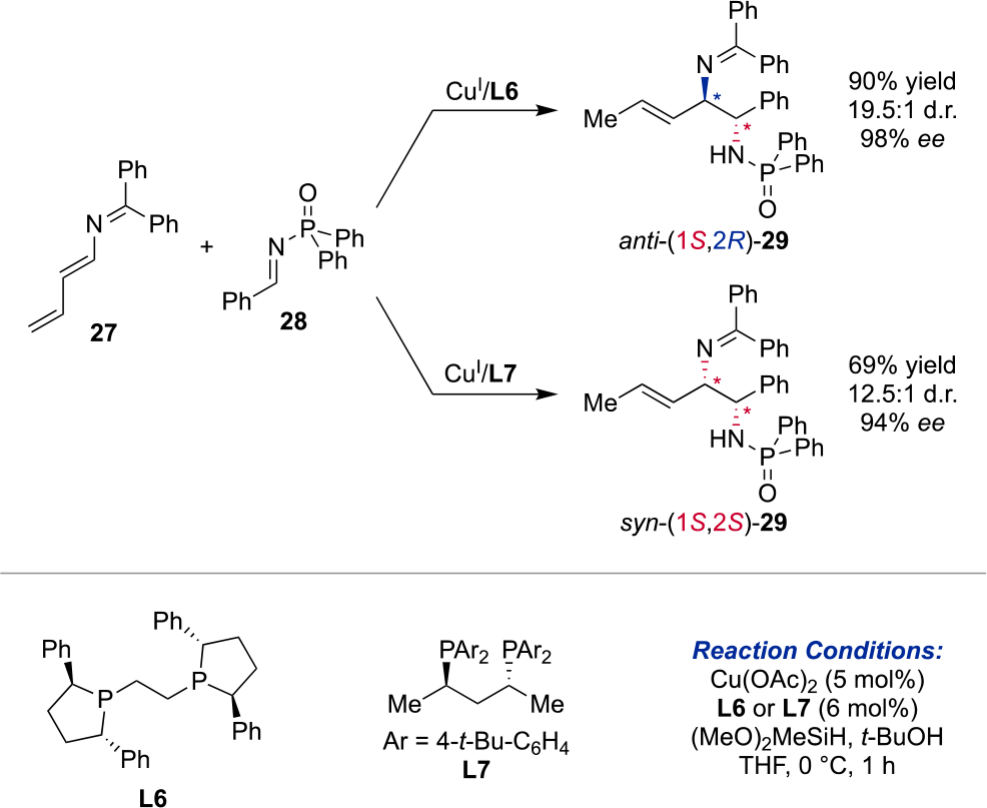
Cu-Catalyzed Coupling of 2-Azatriene 27 and Imine 28 Leads to Diastereodivergent Control over 1,2-Diamine 29

In the past years, the use of two closely related catalysts was disclosed, in particular for copper-phosphine catalyzed reactions which—depending on the specific ligand structure—are able to selectively form all four possible stereoisomers under catalyst control. As a new platform for Cu^I^/bisphosphine-catalyzed reductive couplings with imines, the Malcolmson group introduced a new Umpolung reagent, 2-azatriene, to synthesize allylic amines (Scheme 6).^45^ Utilizing **L6** as the ligand in combination with Cu(OAc)_2_, enantioenriched *anti*-diamines were obtained with exquisite diastereocontrol. Under otherwise identical conditions, the ligand **L7** promotes the formation of *syn*-diamines with 94% *ee* and >20:1 d.r. Similar oxidative borylative couplings were already disclosed by the Oestreich^46^ and Kanai groups,^47^ observing the same effect in ligand alterations.

**Scheme 7:**
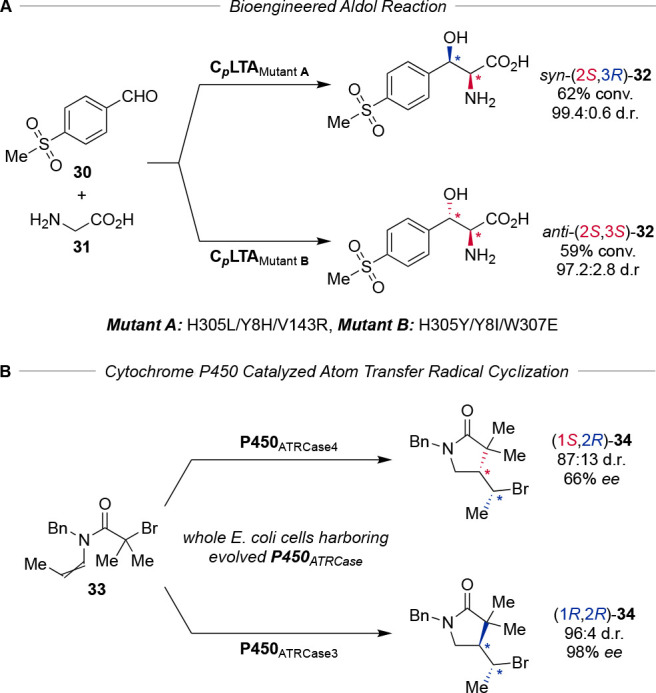
Directed Evolution Enables Diastereodivergent Biocatalysis (A) Aldol addition and (B) atom transfer radical cyclization.

With biochemical engineering, the design and optimization of biocatalyst structures have facilitated the emergence of new biocatalytic methods for the diastereodivergent control over two stereocenters.^48^ By directed genetic evolution and the continuously developing artificial networks for annotating sequences, which consequently enables faster identification and tailoring of enzymes, the availability of highly specialized biocatalysts steadily increases.^49^ An illustrative example of the potential of enzymes in diastereodivergent reactions is the l-threonine aldolase (LTA) catalyzed aldol reaction between 4-methylsulfonylbenzaldehyde (**30**) and glycine (**31**) to forge a high-value precursor **32** for antibiotics (Scheme 7A).^50^ A specialized directed evolution strategy and steered molecular dynamics (SMD) simulation indicated that the enzyme possesses two different substrate access tunnels—the *anti*- and the *syn*-tunnel—which enable inverted diastereoselectivities.

Inspired by the elemental redox-properties of first-row transition metal cofactors, Yang and co-workers disclosed engineered cytochromes P450 to achieve excellent levels of selectivity in diastereodivergent atom transfer radical cyclizations (Scheme 7B).^51^ The induced transformation assembles *anti*-lactams (1*R*,2*R*)-**34** in 96:4 d.r. and 98% *ee*, while the corresponding *syn*-lactam is accessible in 87:13 d.r. and 66% *ee*. The evolvable platform of metalloenzymes could serve as a starting point for the exploration of new-to-nature reactivity, exploiting the accessible chemical space in diastereodivergent catalysis.

## 4 Dual-Catalytic Methods for the Simultaneous Diastereodivergent Control over Two Stereocenters

### 4.1 Sequential Dual Catalysis

**Scheme 8:**
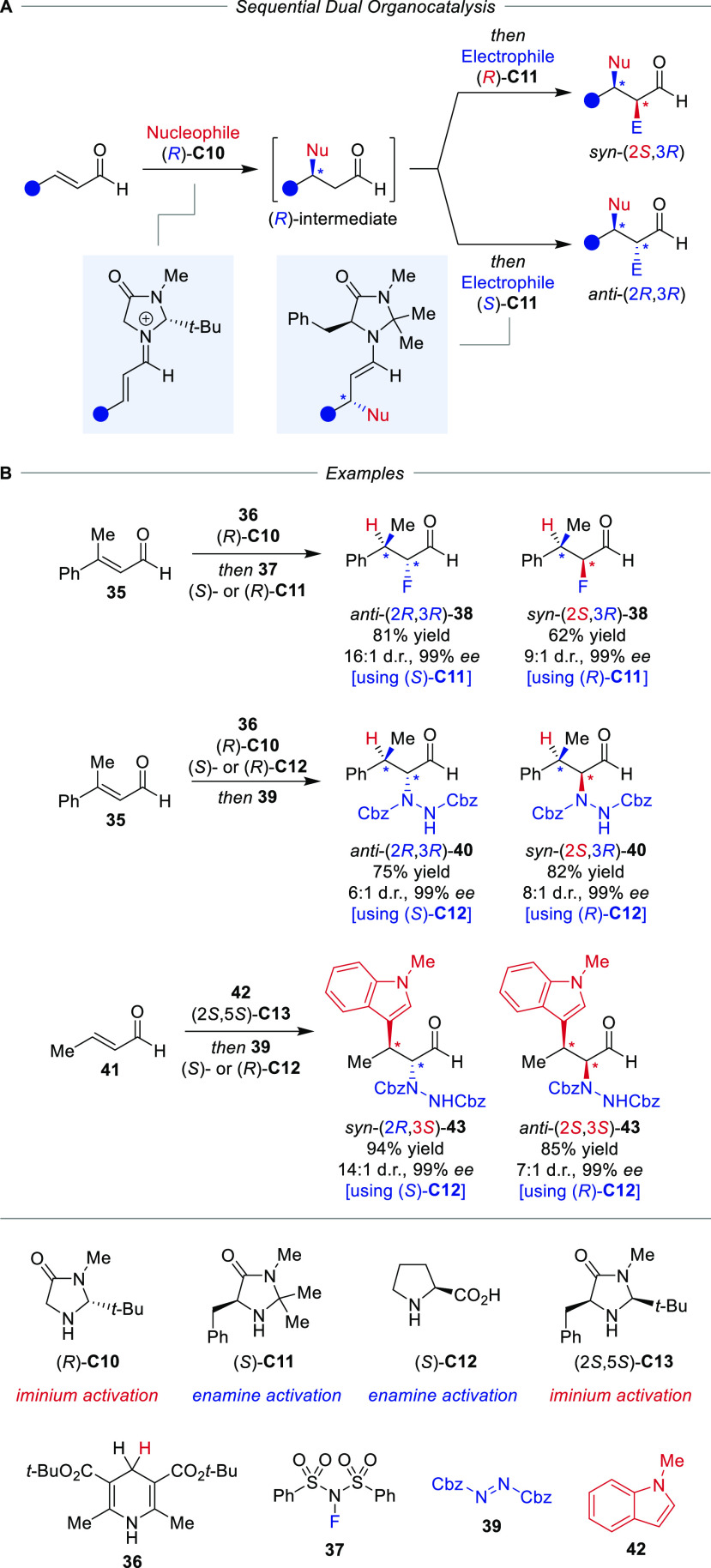
Diastereodivergent Sequential Iminium–Enamine Catalysis

Predictability of the stereochemical outcome of a diastereoselective reaction, in which two stereogenic units are formed in a single step or two consecutive reactions, is—in the ideal case—possible with well-designed dual catalytic systems. Closely related to the stepwise control over stereogenic units, two enantioselective reactions can be combined in one pot to set the configuration of two stereogenic elements. Importantly, the reactivity of the product of the first reaction should be orthogonal to the reactivity of the starting material, so it can directly undergo the ensuing stereoselective reaction, i.e. contrasting iterative diastereodivergence, no workup or preparation of the substrate for the next step is performed. In sequential dual catalysis, a catalyst governs an enantioselective reaction yielding an intermediate which is subsequently activated by a second catalyst for a second reagent, both of which are added once the first step is complete. While this strategy does not require compatibility between the reagents of the individual steps, it still demands partial orthogonality of the two catalysts, as both catalysts are present in the second step. However, compared to relay and synergistic catalysis (*vide infra*) the requirements are yet lower, as only the byproducts of the reagents must be compatible and the catalyst of the first step must not be active in the second step. Approaching relay catalysis, i.e., when very high cycle-specificity between the catalysts is given, both catalysts can be present from the outset of the reaction with the second reagent being added after consumption of the first one. These requirements can be met by dual catalyst systems comprising two secondary amine catalysts, as it was shown by the MacMillan group (Scheme 8A).^38,39^ Activation of an enal by iminium formation with a secondary amine catalyst furnishes after conjugate addition of a nucleophile an α,β-saturated aldehyde. This can be converted in the presence of a secondary amine with an electrophile through enamine activation. If these two transformations are to be combined in one pot under diastereodivergent conditions, two complementary amine catalysts need to be used as inversion of the stereoselectivity of the second step has to be possible, and cycle-selectivity, i.e., the orthogonal activation as iminium ion and enamine by the two distinct catalysts, is necessary. This goal was achieved in the overall hydrofluorination of enal **35** with Hantzsch ester **36** as hydride donor and fluoro sulfonimide **37** as electrophilic fluorine source under control of two chiral secondary amine catalysts **C10** and **C11** (Scheme 8B). When the scope was expanded, the combination of enamine and iminium catalysts **C10** and **C12**, respectively, even allowed for the presence of both catalysts from the outset of the reaction in the overall hydroamination of enal **35**, demonstrating the exceptional cycle-specificity of the two catalysts employed. Diastereodivergence, which still showed clear match/mismatch behavior, was achieved not only in formal hydrofunctionalizations, but also for the addition of other nucleophiles, such as indole **42** or protected hydroxylamine with other electrophiles, e.g. nitrosobenzene. Notably, in all examples, the *ee*’s of the products were very high and in agreement with the Horeau principle.

**Scheme 9:**
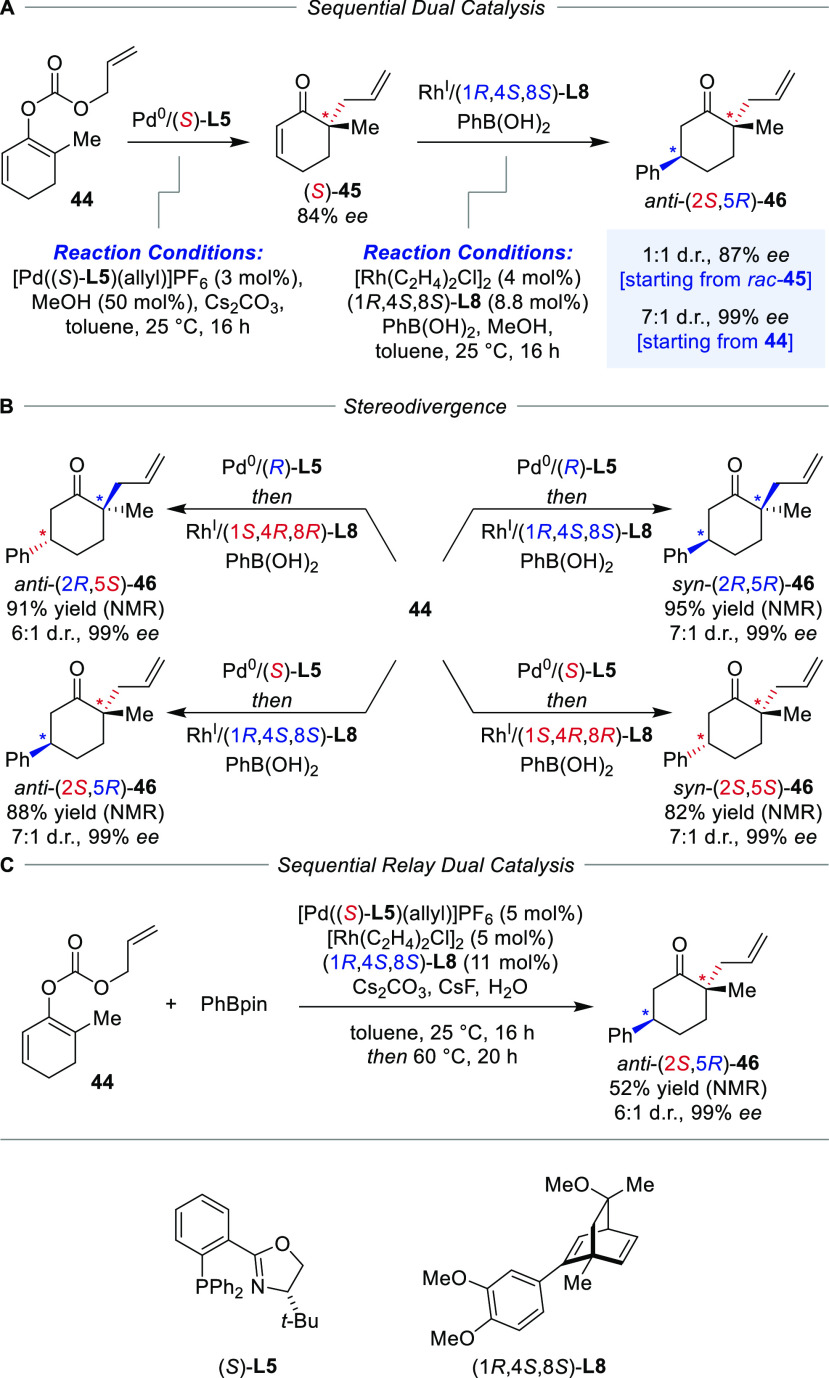
Sequential Dual Catalysis Using Two Chiral Metal Complexes for Stereoselective Allylation and Conjugate Addition

Diastereodivergent sequential dual catalysis was also reported with two transition metal-based chiral catalysts by Lautens and co-workers (Scheme 9).^52^ In contrast to the previously discussed iminium/enamine activation of enals, no match/mismatch behavior was observed as a consequence of the generation of remote noninteracting stereocenters. Namely, allyl enol carbonate **44** was transformed into ketone **46** carrying an α- and a δ-stereocenter by a sequential Pd-catalyzed decarboxylative allylation^53−55^ and Rh-catalyzed conjugate addition^56−58^ to intermediate **45**. In the first step, a Pd^II^-precatalyst with the PHOX ligand **L5** already bound to the metal center was employed to avoid coordination of the consecutively added Rh-catalyst, which would render it inactive. However, the use of the Pd^II^-precatalyst required the addition of Cs_2_CO_3_ and methanol to generate the active Pd^0^-species. Conducting the allylation with PhB(OH)_2_ in an isolated reaction, enone (*S*)-**45** was obtained with an 84% *ee* (Scheme 9A). The installation of the second δ-stereocenter was unaffected by the configuration of the α-stereocenter, as the reaction of *rac*-**45** revealed, yielding a d.r. of 1:1 with 87% *ee* (Scheme 9A). Thus, not expecting a substrate–catalyst match or mismatch situation, combining the two individual steps in a sequential dual catalytic protocol produced *anti*-(2*S*,4*R*)-**46** in the expected 7:1 d.r. and high 99% *ee* in accordance with the Horeau principle. Reacting the same reactants with all catalyst permutations yielded all four stereoisomers in similar yields and enantio- and diastereoselectivities, underlining the low level of substrate bias in these transformations (Scheme 9B).

Aiming for relay dual catalysis, the compatibility between the reactants was tested and the presence of PhB(OH)_2_ resulted in a low conversion in the stereoselective allylation, whereas PhBpin was tolerated at room temperature but required a temperature of 60 °C to react in the second step. In a sequential relay catalytic reaction, all catalysts and reagents were present from the outset, and orthogonal reactivity was achieved by implementing a temperature gradient from 25 to 60 °C to promote the conjugate addition step (Scheme 9C). However, this protocol gave rise to a slightly decreased diastereoselectivity, a reduced yield of 52% and a more challenging purification of ketone product **46**.

### 4.2 Relay Catalysis

**Scheme 10:**
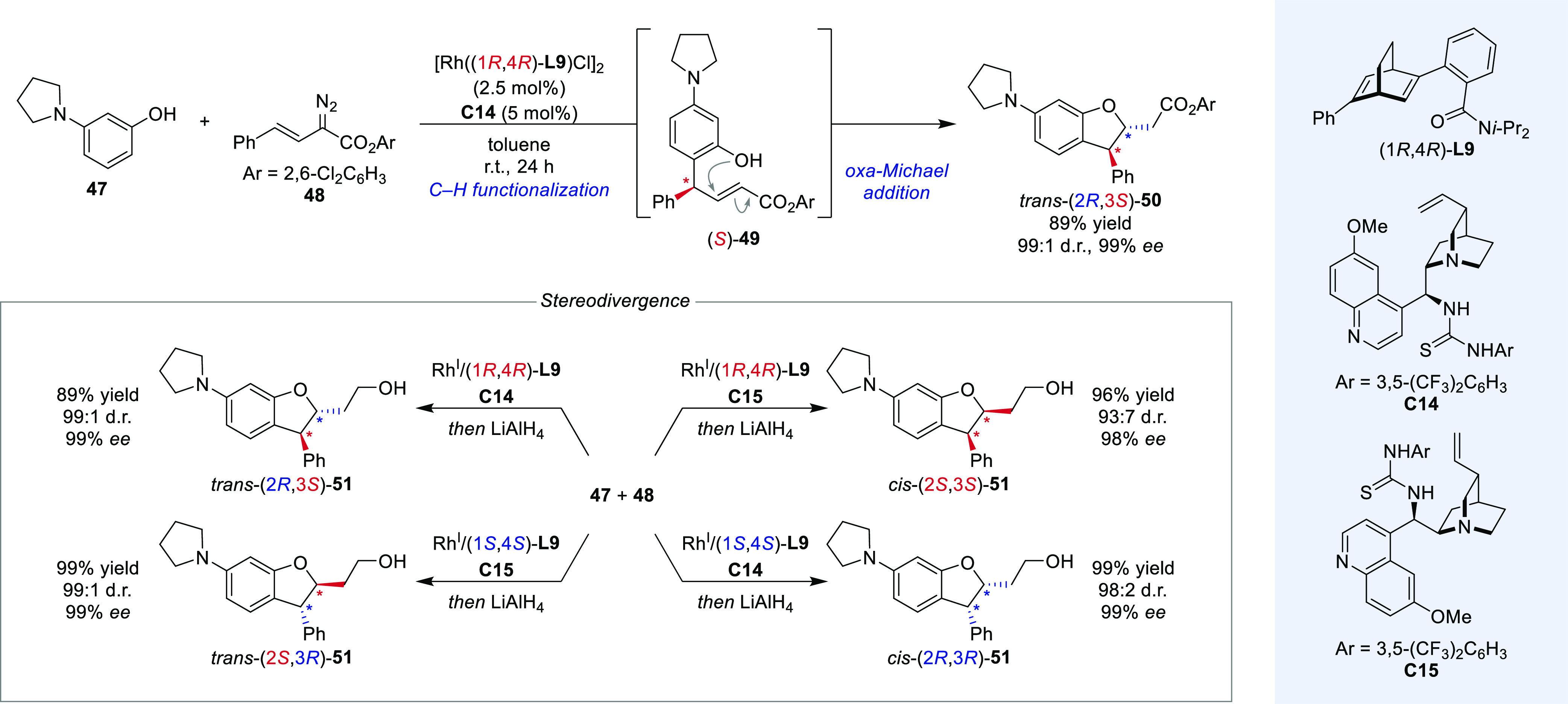
Relay Catalysis Cascade in the Stereodivergent C–H functionalization/Oxa-Michael Addition between 47 and 48

Although sequential and relay catalysis share the same general mechanism (Figure 1C), they differ strongly in a very practical aspect. While in relay catalysis all reaction components are present from the outset of the reaction, imposing high demands on the compatibility between them, in sequential catalysis, the reagents and catalysts for the second step are added after completion of the first one. The last example of the previous section illustrates the challenges associated with these one-pot reactions, and although the incompatibility of PhB(OH)_2_ with the Pd-catalyzed reaction step was circumvented, the one-pot reaction led to deteriorated yield and selectivity. Nevertheless, relay catalysis was shown to deliver diastereodivergence in a C–H functionalization–oxa-Michael addition cascade starting from activated phenol **47** and arylvinyldiazoacetate **48** (Scheme 10).^59^ In the first step of this reaction sequence, the Rh-catalyst activates diazo compound **48** as electrophilic vinyl carbene complex followed by enantioselective conjugate addition of the activated arene in an S_E_Ar-type mechanism.^60^ Hydrogen transfer to rhodium under rearomatization and subsequent reductive elimination yielded α,β-unsaturated ester **49** in enantioenriched form. Bifunctional thiourea catalyst **C14** then guided the intramolecular oxa-Michael addition to one diastereomer of dihydrobenzofuran **50** exerting control over the second stereocenter. Notably, the intramolecular nature of this second reaction step lowers the requirements for compatibility, because no additional reagent needs to be tolerated. To invert the configuration of the stereocenter formed in the second step, pseudoenantiomeric thiourea **C15** was employed and the set of all four conceivable stereoisomers was obtained in excellent diastereo- and enantioselectivities (93:7–99:1 d.r., 99:1 e.r.). Because of its efficiency, diastereodivergent relay catalysis with a wider range of compatible catalyst classes and reaction manifolds may be anticipated in the future.

### 4.3 Synergistic Catalysis

**Scheme 11:**
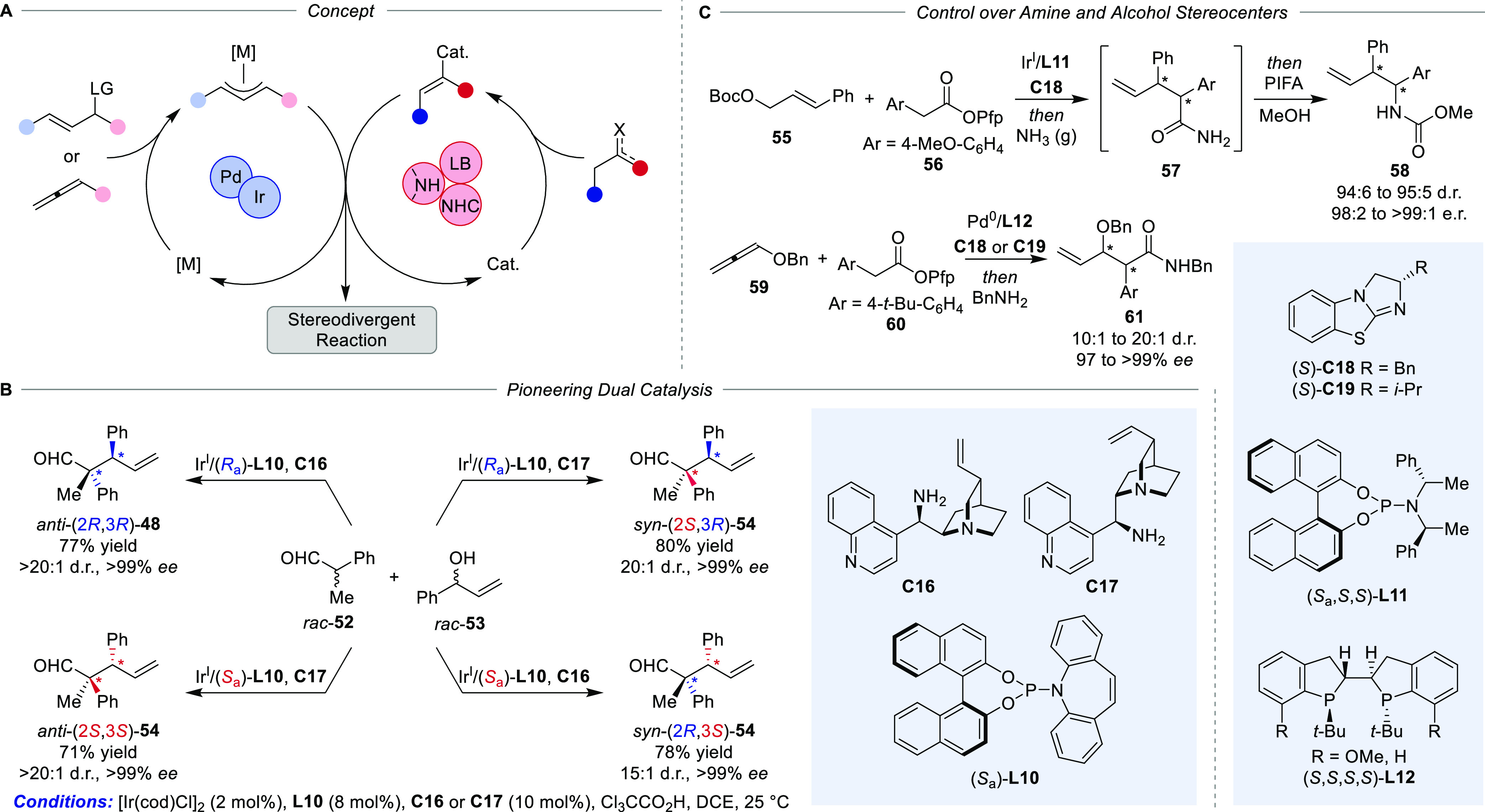
Metal–Organo Synergistic Dual Catalysis for Diastereodivergent Reaction Control (A) Concept, (B) pioneering study on the α-allylation of aldehydes, and (C) diastereodivergent synthesis of amines and alcohols. Pfp = perfluorophenyl.

Synergistic dual catalysis, in which two reactants are activated by two distinct catalysts, has the potential to avoid the substrate–catalyst mismatch situations of sequential catalysis since two stereogenic units are controlled simultaneously. The most widely studied reaction class within diastereodivergent synergistic dual catalysis is the allylation of enolates or equivalents thereof (Scheme 11A). A potential strategy to address the challenge of independent electrophile and nucleophile activation is the combination of two fundamentally different catalysts—such as metal-based and organocatalysts—therefore exploiting significant differences in chemical affinity and reactivity. In this context, the identification of the suitable combination of noninterfering catalysts is crucial for the stereocontrolled assembly of two distinct stereogenic elements overcoming potential intrinsic matched/mismatched scenarios.

An important breakthrough employing this strategy was disclosed by Carreira and co-workers who designed a dual catalytic system for the diastereodivergent α-allylation of branched hydratropaldehyde,^61^ which was later expanded to linear,^62^ α-amino- and α-hydroxy-substrates (Scheme 11B).^63^ Imperative for the success of this system are the isolated catalytic cycles functioning alongside. While the chiral amine catalyst sets the α-stereocenter, the Ir-phosphoramidate complex controls the configuration of the vicinal β-stereocenter. In a subsequent study by the Dong group, allylation of α-branched aldehydes was realized by Rh-catalyzed alkyne hydrofunctionalization.^64^ A major difference in this design lies in the generation of the electrophilic allyl species. Contrary to prevailing Ir- and Pd-catalysts, the employed Rh-catalyst underwent insertion, β-hydride elimination, and reinsertion into the intermediate allene, allowing the regioselective formation of a Rh-π-allyl complex. Activation of the aldehyde counterpart was accomplished by enamine formation through Jacobsen’s amine.

Beside enamine catalysis, activation of the nucleophile was furthermore realized with chiral Lewis base catalysts,^65,48^ allowing for the diastereodivergent one-pot synthesis of homoallylic amines when coupled with a subsequent Hofmann rearrangement.^66^ Similar to the Carreira group’s work, employing an Ir/phosphoramidate catalyst resulted in the formation of a chiral metal-π-allyl species, whereas Birman’s benzotetramisole **C18** was used in synergistic manner to form chiral C1-ammonium enolates from perfluorophenylesters **56** (Scheme 11C). The allylic alkylation provided intermediate primary amides **57** after an *in situ* reaction of the perfluorinated ester products with gaseous ammonia. A subsequent stereospecific Hofmann-type rearrangement mediated by PIFA and interception of the resulting isocyanates with an appropriate alcohol gave the corresponding carbamate-protected branched homoallylic amines in almost enantiopure form. Permutation of the catalyst configurations consequently provided stereodivergent access to all feasible stereoisomers of **58** with similar excellent stereoselectivity (94:6 to 95:5 d.r., 98:2 to >99:1 e.r.). This synthetic strategy was more recently further developed into a diastereodivergent aldol-type coupling starting directly from alkoxyallenes **59** and perfluorophenyl esters **60** under cooperative Pd/chiral Lewis base catalysis.^67,68^ Additionally, the same elementary concept was further employed for the diastereodivergent coupling of 1,3-dienes and perfluorophenyl acetates.^69^

The concept of combining the transition-metal based catalysts with a second characteristically distinct organocatalyst was further expanded to include NHC catalysis for the diastereodivergent synthesis of α,β-disubstituted γ-butyrolactones.^70^ Furthermore, a C(sp^3^)–C(sp^3^) bond-forming coupling between 1,3-dienes and oxindoles represents a rare example of dual-catalytic stereodivergent syntheses based on the merger of palladium and ion-pairing catalysis.^71^

**Scheme 12:**
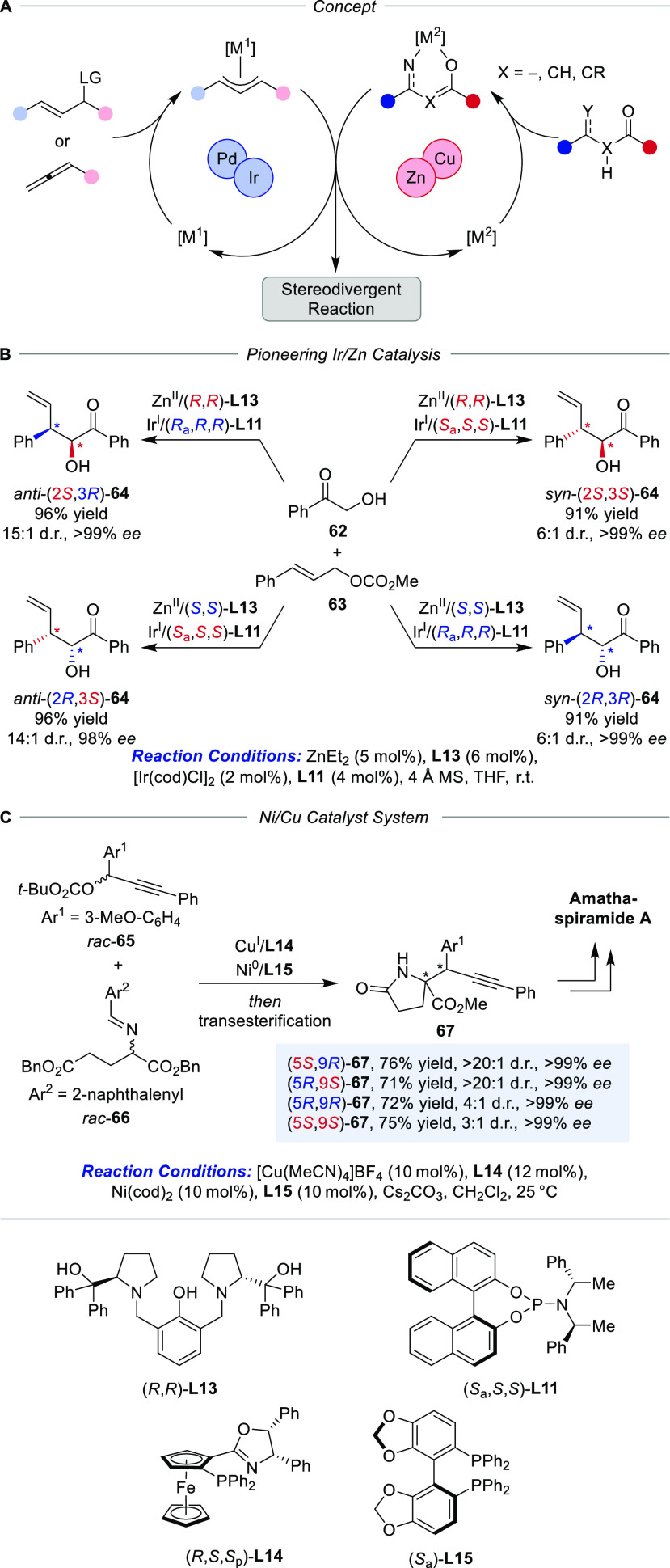
Metal–Metal Synergistic Dual Catalytic Systems for the α-Allylation of Carbonyl Compounds (A) Concept, (B) pioneering Zn/Ir-catalysis, and (C) application in the synthesis of natural products.

Despite the discernible advantages of combining transition metal with organocatalysts, bimetallic catalysis has emerged as a valuable concept for addressing diastereodivergent allylations.^16^ While the allyl component is introduced to the catalytic cycle in the form of an allyl Pd- or Ir-complex generated from an allene,^72,73^ allyl alcohol,^74^ ester,^75,76^ enyne,^77^ diene,^78^ carbonate,^79−81^ or 2-vinyloxirane,^82^ the enolate compound is activated by a second metal catalyst with distinct substrate affinity and consequently chemoselectivity. To achieve efficient stereocontrol with Zn- and Cu-enolates and to govern the configuration (*cis/trans*) of the enolate component, the substrate needs to bear a donating group–typically an imine–to chelate the metal center (Scheme 12A). A chiral ancillary ligand, together with the chiral ligand on the allyl complex, then governs the stereochemical outcome of the allylation reaction based on steric repulsion.

A pioneering bimetallic synergistic catalyst system for diastereodivergent catalysis was disclosed by Zhang and co-workers in 2016 consisting of a chiral Ir-catalyst and Zn/**L13**-enolate complexes for the stereodivergent α-allylation of unprotected α-hydroxyketones (Scheme 12B).^81^ By alteration of the ligand configurations, complete stereodivergence with excellent stereoselectivity for all conceivable stereoisomers as major products was possible (6:1 to 15:1 d.r., 98 to >99% *ee*). Here, the highly specific affinity of the employed ligands for Ir and Zn, respectively, is crucial for the realization of this concept, as ligand exchange would eventually lead to diminished selectivities. Through the years, the combination of Pd or Ir–for the formation of electrophilic π-allyl species–and Cu or Zn–for nucleophilic enolate formation–proved to be the most reliable strategy for achieving diastereodivergence. Commonly, the choice of transition metals can be traced back to their inherent and distinct reactivity profiles. Different from Pd-catalyzed allylations, the Ir-variant typically results in branched products^83^ and the differing diastereo- and regioselectivities were recently investigated in a detailed computational study.^84^ The potential chemical space accessible by means of synergistic bimetallic catalysis spans from simple α-allylations of carbonyls to the synthesis of nonproteogenic α-amino acids bearing vicinal quaternary or tertiary stereocenters. The formation of these valuable compounds was realized by means of Cu/Ir-synergistic catalysis starting from aldimine esters and suitable π-allyl complex precursors.^85^ The full set of conceivable stereoisomers could be obtained by alteration of the configuration of the used phosphoramidate ligands for Ir and Phosferrox ligands for Cu under excellent catalyst stereocontrol. More recently, Guo and co-workers developed a synergistic bimetallic system based on Ni and Cu catalysis for the propargylation of aldimine esters.^86^ Based on this work, this concept was extended to realize the programmed stereodivergent total synthesis of amathaspiramide D (Scheme 12C).^80^ This study highlights the progress of synergistic metal–metal dual catalysis within less than a decade.

**Scheme 13:**
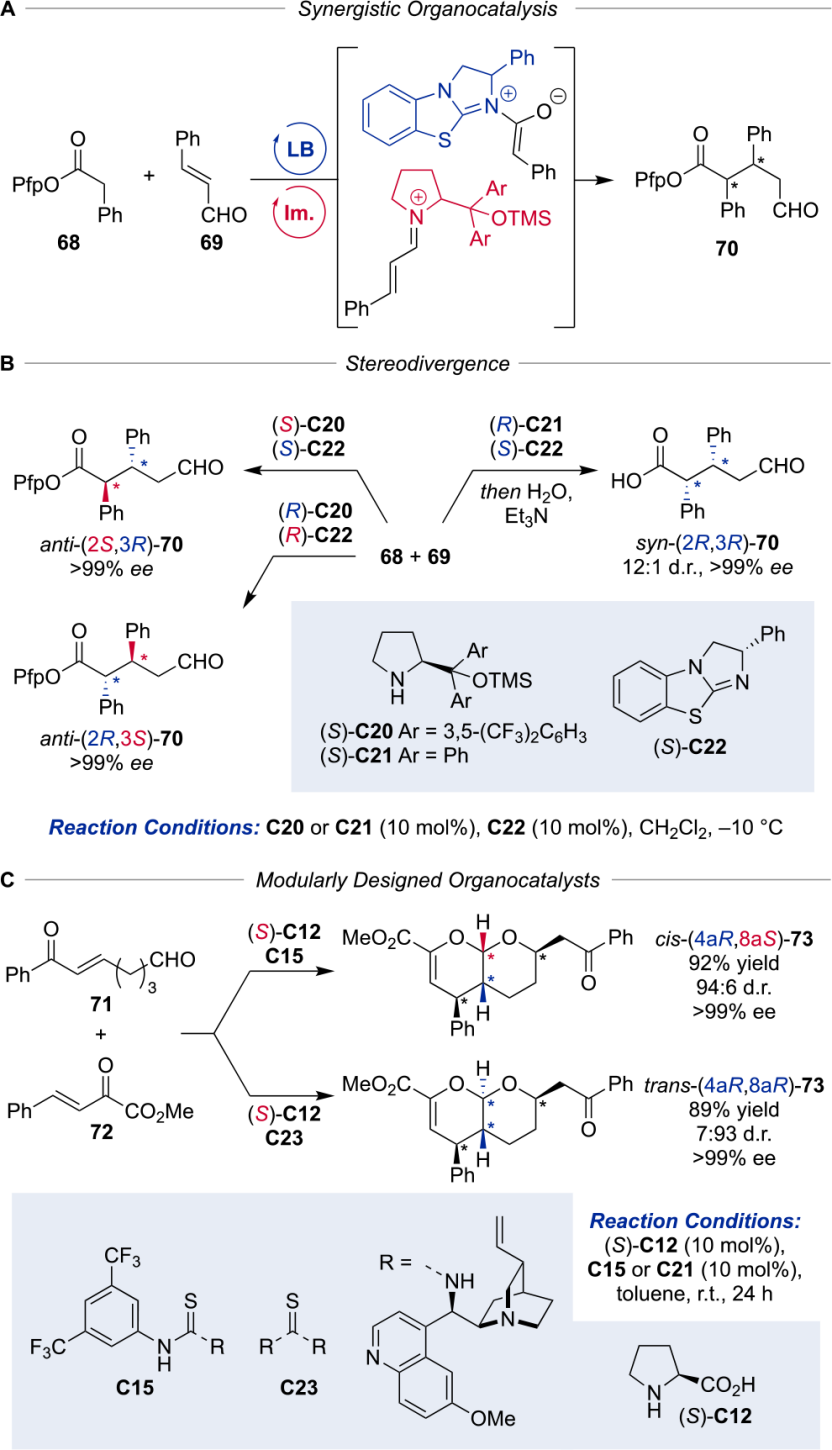
Organo–Organo Synergistic Dual Catalysis Enables Stereodivergent Michael Addition PfP = perfluorophenyl.

While the majority of the present studies profoundly uses the same concept—the generation of highly electrophilic metal-π-allyl species and nucleophilic enolates or equivalents thereof—a solely organocatalytic synergistic catalytic system would be highly desirable to avoid the use of precious metal catalysts for the activation of the allyl component. Despite the advances in organocatalysis, the development of methodologies addressing diastereodivergent dual catalysis is still in its infancy. Dual-organic synergistic catalysis was reported recently by Lee and co-workers employing synergistic Lewis base/iminium catalysis (Scheme 13).^87^ The presented diastereodivergent Michael addition was realized using benzotetramisole **C22** as the chiral Lewis base generating C1-ammonium enolates by the reaction with electron-deficient aryl esters **68** with high facial selectivity.^88^ Combined with highly electrophilic iminium species derived from cinnamaldehyde **69** and Jørgensen–Hayashi amine catalysts **C20** and **C21** respectively, the reaction proved to be highly diastereodivergent forming enantiopure *anti*-diastereomers with excellent selectivities (>99% *ee*, no other diastereomer detected). After further optimization, the *syn-*selective Michael addition was realized furnishing the corresponding enantiopure carboxylic acid aldehydes **70** after hydrolysis, in good diastereoselectivities (ca. 10:1 d.r.). Crucial for the observed diastereodivergence was the high cycle specificity of the established catalysts, which allowed the independent activation of otherwise unreactive aldehyde and ester substrates. The necessity for perfluorinated aryloxide as a leaving group to prevent uncatalyzed racemic background reactions, due to low nucleophilicity, represents a minor limitation of this methodology. Nevertheless, the obtained 1,5-dicarbonyl products proved to be well-suited for subsequent modifications, introducing new ensuing stereocenters.

In a recent study, partially meeting the requirements of divergence, Zhao and co-workers described the synthesis of all four accessible *cis-* and *trans-*fused pyrano[2,3-*b*]pyran derivatives using a tandem inverse electron-demand hetero-Diels–Alder/oxa-Michael reaction catalyzed by modularly designed organocatalysts (MDO) (Scheme 13C).^89^ The ionic interactions between cinchona alkaloid thiourea ammonium and proline-derived carboxylate ions lead to *in situ* formation of MDOs via self-assembly.^90,91^ Those spatially defined catalysts were able to exert high levels of stereocontrol in the outlined reaction and yielded both enantiomers of the *cis-* and *trans*-configured products. Mechanistic studies revealed that the observed inversion of stereoselectivity is consistent with a transitional dynamic kinetic resolution step achieving diastereodivergence. Present studies addressing the realization of a solely organic dual catalytic system are mainly developing into the direction of relay and sequential catalysis. Incompatibility of reactants, catalysts and reactive intermediates are currently retaining progress, emphasizing the major limitations in this field. Since activation modes of different organocatalyst classes are usually too similar, chemoselectivity is a major issue. However, the development of synergistic concepts enabled by organo–organo dual catalysis remains to be further explored to open up manifold new possibilities for diastereodivergent catalysis once more compatible organocatalyst classes are identified.

## 5 Diastereodivergent Catalyst Control in the Synthesis of Atropisomers

**Scheme 14:**
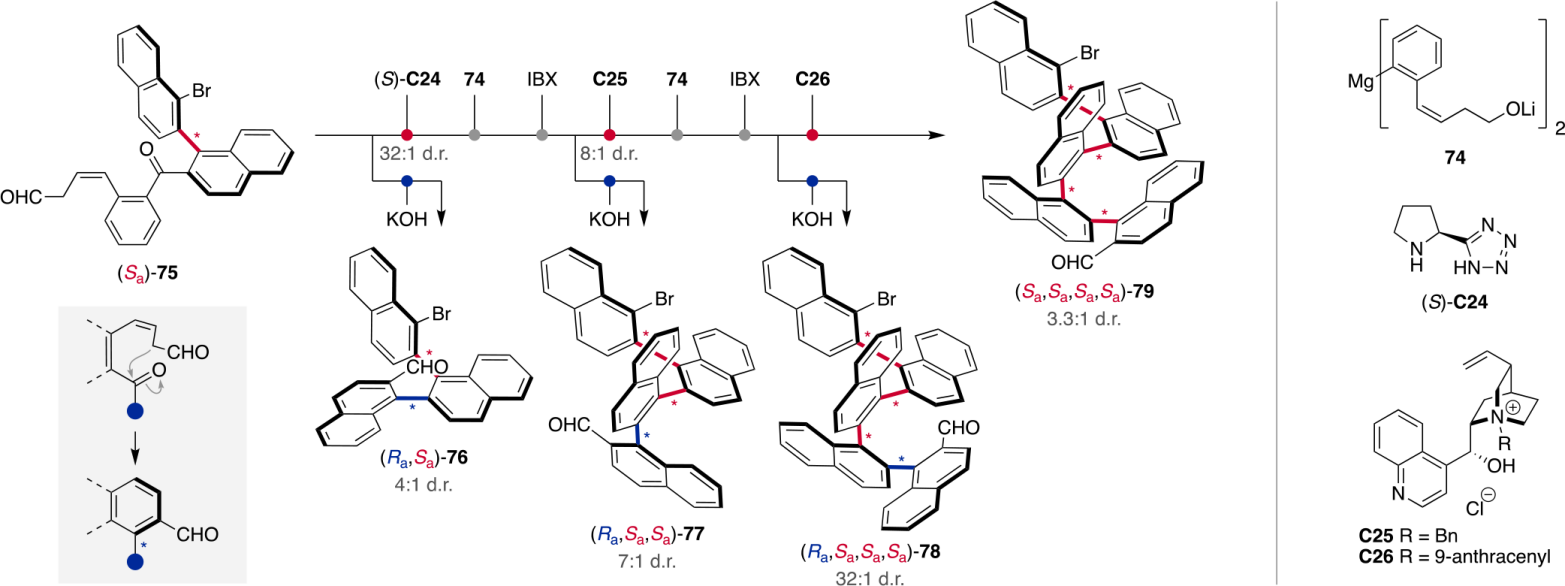
Iterative Diastereodivergent Control over Oligonaphthylenes with Four Stereogenic Axes by Atroposelective Aldol Condensations IBX = 2-iodoxybenzoic acid.

Stereoisomerism is not limited to stereocenters, and the type of stereogenic units which can be prepared in diastereoselective fashion also applies to atropisomers. The main concepts are similar as with stereocenters, since stereogenic axes can be formed iteratively or simultaneously. However, only few methods to diastereodivergently control the configuration of multiple stereogenic axes have been reported to date.^92^ Arene-forming aldol condensation reactions were shown to enable the enantioselective synthesis of atropisomeric binaphthyls and amides.^93,94^ Notably, the aldehyde functionality of the atropisomeric product can be used as a linchpin for the iterative expansion of the system by the addition of organometallic building block **74** (Scheme 14).^95^ After oxidation, another atroposelective aldol condensation gives rise to two diastereomeric products potentially allowing for iterative control over several stereogenic axes leading to oligonaphthylenes. Starting from enantioenriched (*S*_a_)-**75**, which was synthesized by an enantioselective aldol condensation, oligonaphthylenes with up to four stereogenic axes were prepared with diastereodivergence in every cyclization step. While to control the second axis the secondary amine catalyst **C24**, which was also utilized in the enantioselective reaction leading to **75**, could be employed, the use of ion-pairing catalysts was further shown to be suitable for diverting the selectivity in the mismatched case, which was tested in the KOH mediated aldol reaction. Interestingly, the substrate bias increases with growing chain length and overcoming it becomes more challenging, illustrating the requirement for a pronounced catalytic activation. While this iterative approach toward atropisomers with contiguous stereogenic axes required careful optimization of each iteration step to sequentially control the stereogenic axes, their simultaneous formation would grant a more streamlined access to stereochemically complex atropisomers in stereoisomerically well-defined form.

**Scheme 15:**
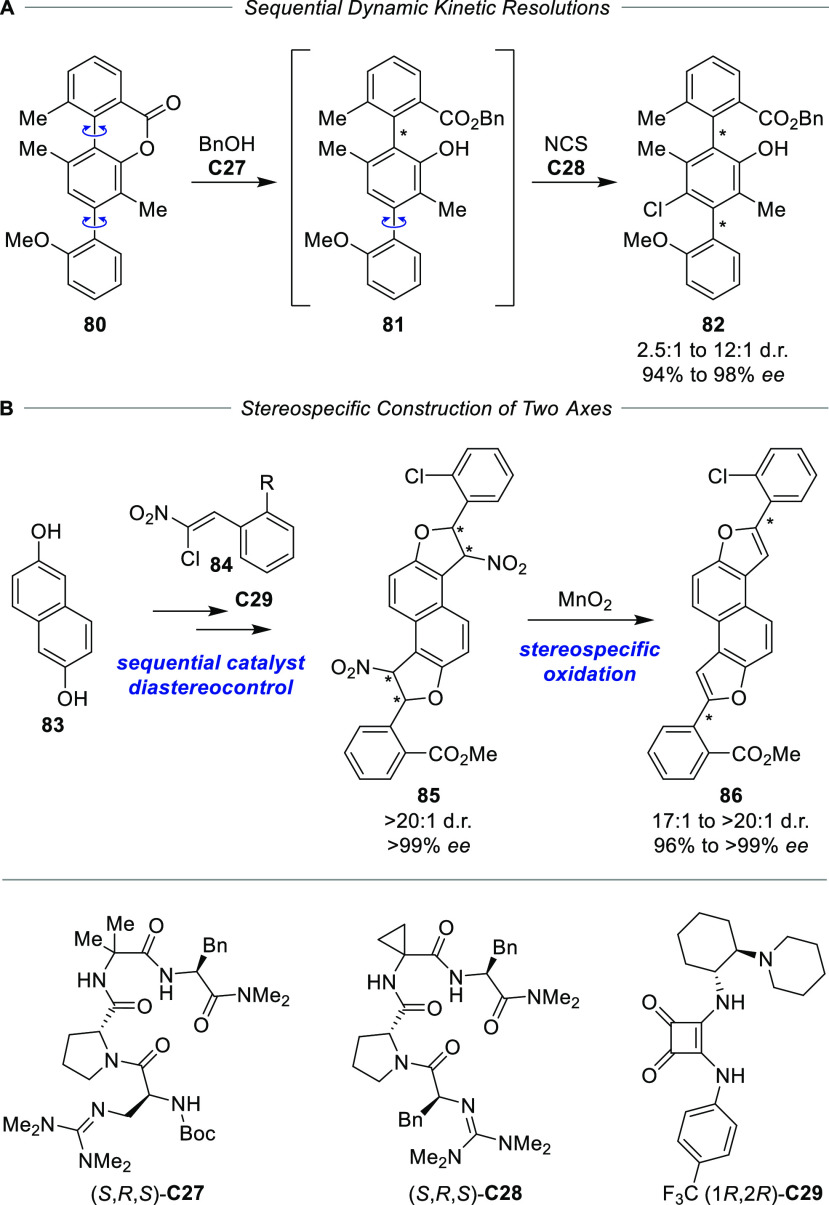
Diastereodivergent Synthesis of Dual Axis Systems Using Sequential Catalyst Control (A) Sequential double kinetic resolution and (B) diastereodivergent formation of stereocenters and stereospecific oxidation to the atropisomeric system. NCS = *N*-chlorosuccinimide.

A pronounced substrate bias was also found in the formation of two stereogenic axes in *para*-position of a benzene ring in **82** by sequentially applying two peptide-catalyzed dynamic kinetic resolutions (Scheme 15A).^96^ While the first axis was formed by the opening of a Bringman lactone motif,^97^ the second one was constructed by chlorination of the central aromatic ring *ortho* to the rotationally dynamic axis in intermediate **81**.^98^

Two more remote axes were controlled making use of the stereospecific transformation of configurationally defined stereocenters into stereogenic axes upon oxidation (Scheme 15B).^99^ Thereby the overall diastereodivergence for the atropisomeric products **86** was the result of the diastereodivergent construction of the stereocenters in intermediate **85** by bifunctional squaramide–tertiary amine **C29** catalyzed stepwise cyclization between naphthalene-2,6-diol (**83**) and two (*Z*)-(2-chloro-2-nitroethenyl)arenes **84**.

**Scheme 16:**
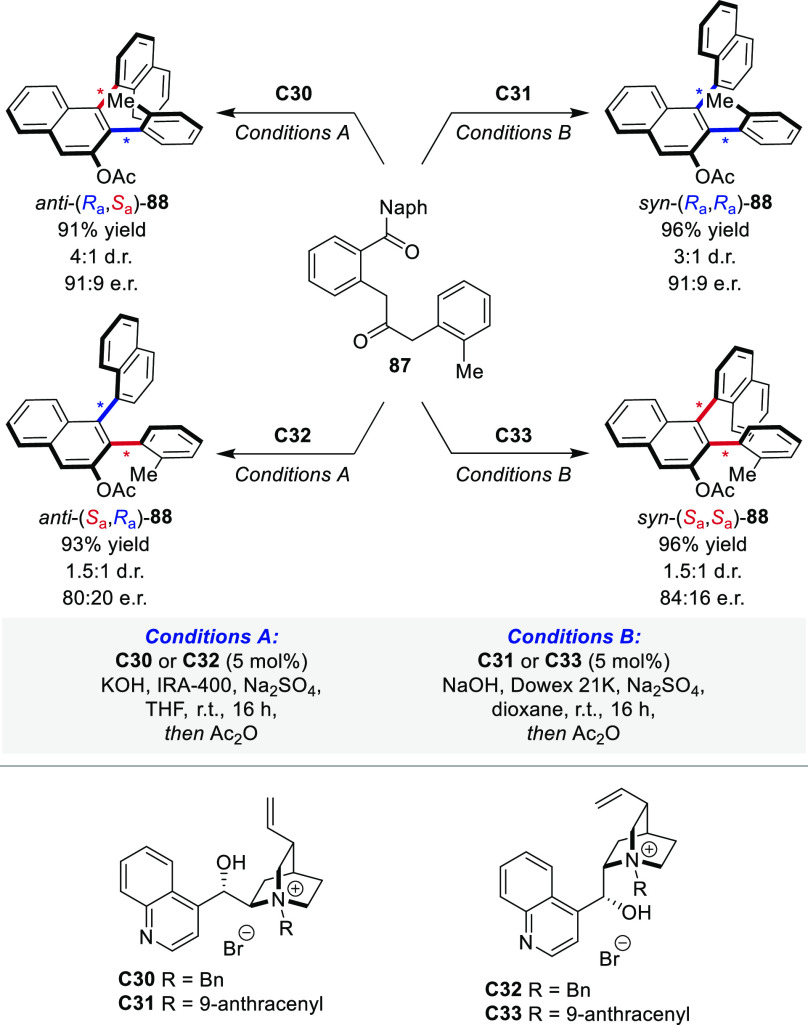
Diastereodivergent Synthesis of a Dual Axis System 88 by Arene-Forming Aldol Condensations

While in the previous examples the stereogenic axes are formed in two independent steps, the formation of two contiguous axes in **88** was realized in a single step from diketone substrate **87** (Scheme 16).^100^ Guided by the polyketide pattern,^101^ two consecutive stereogenic axes were established by the formation of a central β-naphthol as aromatic unit. Starting from the same diketone substrate, the catalytically controlled formation of the two-axis systems was viable by simultaneously addressing both consecutive stereogenic units. The use of cinchona alkaloid-based ion-pairing catalysts combined with alkali metal hydroxides allowed the differentiation of all four feasible reaction pathways, exhibiting complete stereodivergence with high to excellent enantioselectivities for all conceivable atropisomers as major products. The overall selectivity for ion-pairing catalysts derived from cinchonine (**C30** and **C31**) was higher than their pseudoenantiomeric congeners **C32** and **C33**. This is explained by the pseudoenantiomeric nature of the catalysts, which results in a different catalytic reaction environment and impacts the substrate organization. Interestingly, also the *syn*-configured diastereomers with significantly increased steric interactions could be synthesized with high selectivity, allowing the proximity of *ortho*-substituent guided by catalyst control.

**Scheme 17:**
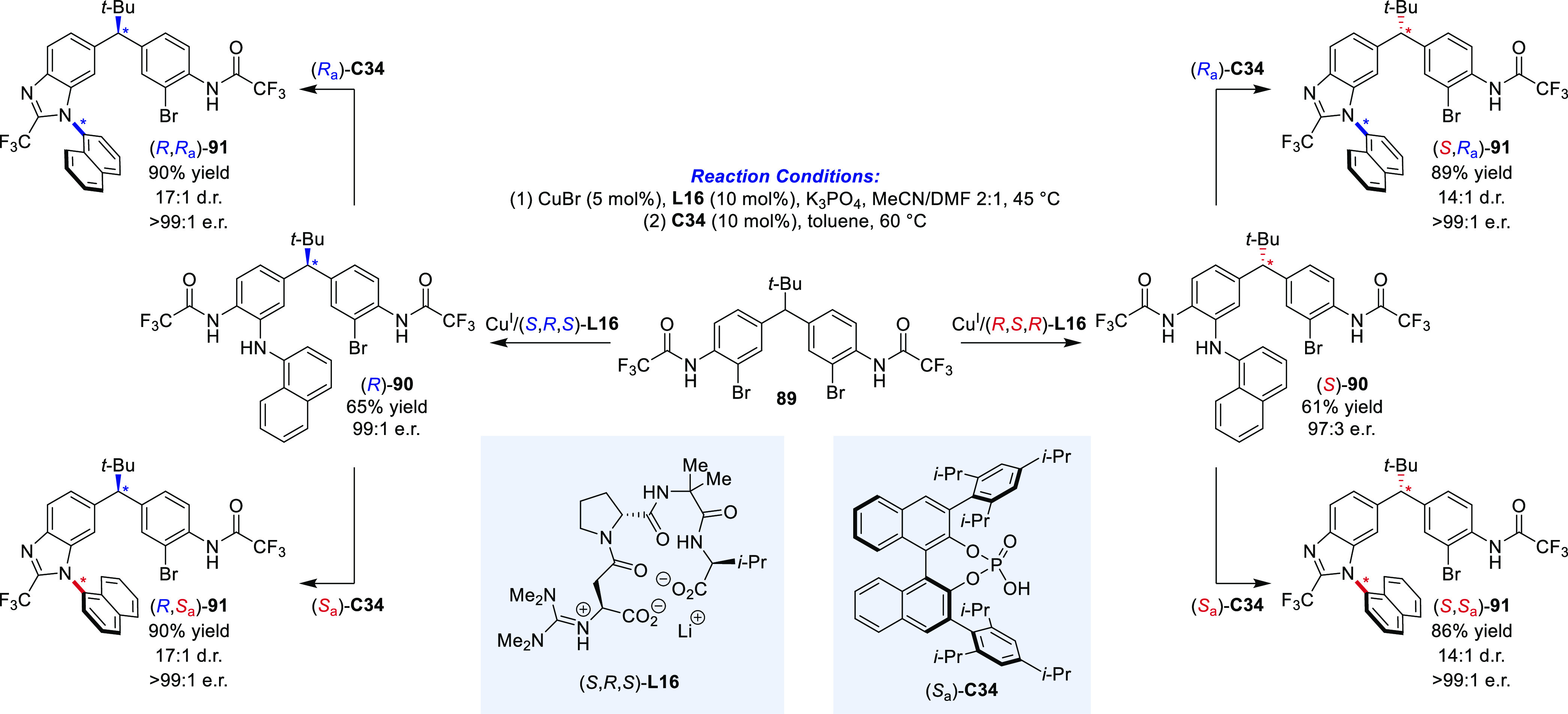
Stepwise Diastereodivergent Construction of a Tertiary Stereocenter and a Stereogenic C–N Axis

**Scheme 18:**
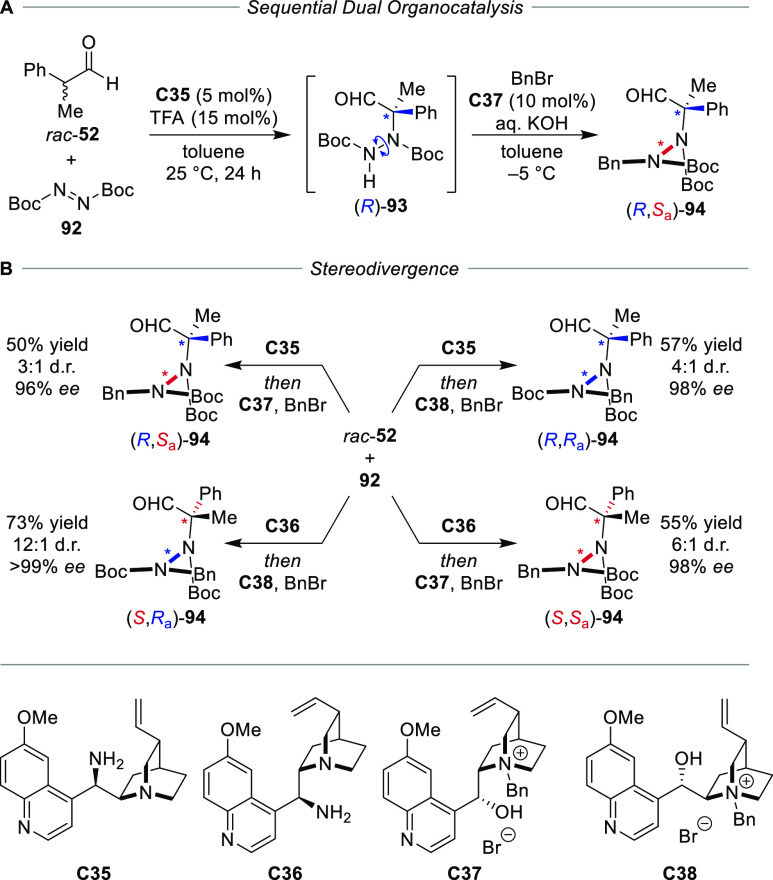
Sequential Dual Catalysis for the Stereodivergent Control over Atropisomeric Hydrazides with a Quaternary Carbon Stereocenter

Expanding the scope of diastereodivergent atroposelective reactions from C–C axes to rotationally restricted C–N bonds, the Miller group combined a tertiary carbon stereocenter with an atropisomeric 1-naphthalenylbenzimidazole (Scheme 17). In a first step, the configuration of the stereocenter was defined by desymmetrization of **89** by an enantioface-differentiating C–N cross-coupling reaction. Thereby, using a copper(I)/peptide **L17** catalytic system, the respective enantiomeric intermediates **90** were obtained with excellent enantioselectivities. Approaching the diastereodivergent construction of the remote stereogenic C–N axis, **90** was subjected to chiral phosphoric acid **C34** promoted dehydrative cyclization, selectively yielding all four stereoisomers by switching the configurations of the peptidyl copper catalyst and **C34**. The high diastereomeric ratios were accompanied by an amplification of the enantiomeric excess. While in this example the stereocenter and the stereogenic axis were remote, a later study by Bencivenni and co-workers showed the diastereodivergent construction of the stereogenic N–N axis of a hydrazide which was substituted with a quaternary carbon stereocenter (Scheme 18).^102^ By stepwise increasing the rotational barrier, the configurations of the two stereogenic elements were set independently in a sequential dual catalytic reaction. Controlled by amine catalyst **C35**, the stereoconvergent amination of racemic aldehyde **52** with azodicarboxylate **92** resulted in rotationally dynamic hydrazide (*S*)-**93** with the configuration of the carbon stereocenter established. Catalyst controlled *N*-alkylation with BnBr under basic phase transfer conditions furnished (*S*,*R*_a_)-**94** with the configuration of the stereogenic axis controlled by catalyst **C37**. Employing pseudoenantiomeric catalysts **C35**/**C36** and **C37**/**C38**, all four stereoisomers could be selectively accessed with diastereoselectivities of up to 12:1 d.r. (96% to >99% *ee*).

## 6 Diastereodivergence for Higher-Order Stereogenicity

**Scheme 19:**
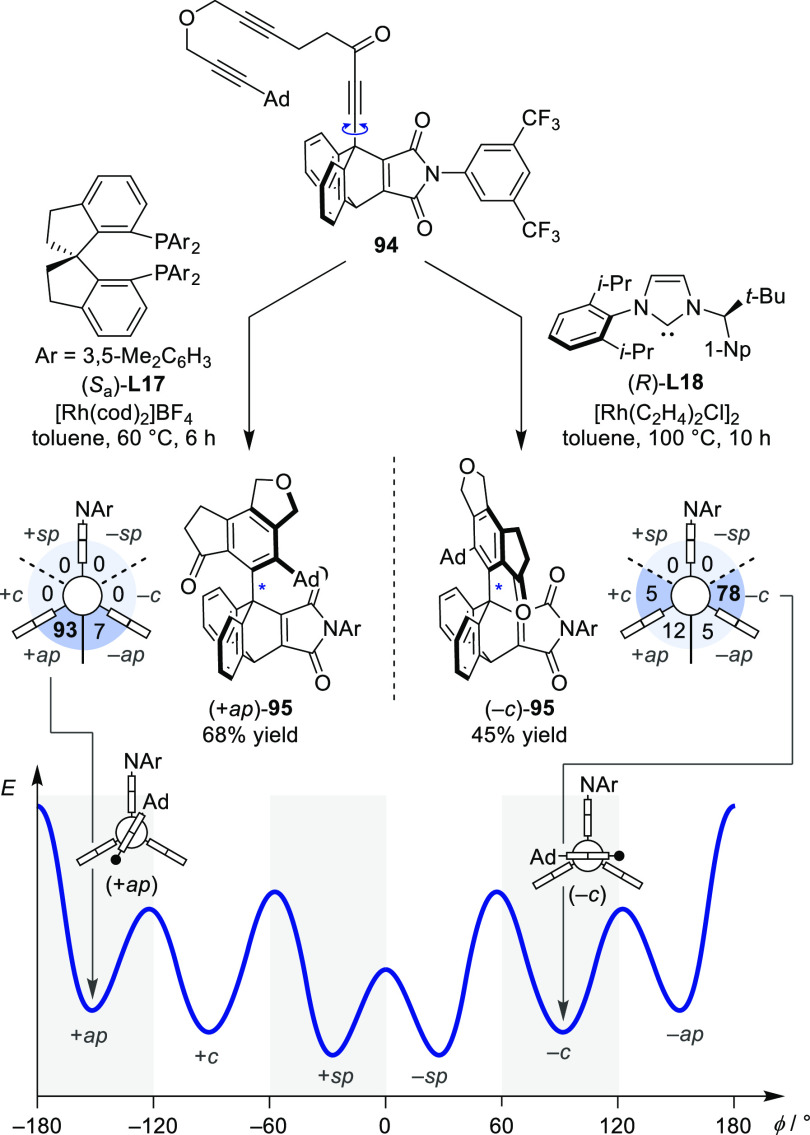
Diastereodivergent and Enantioselective Arene Formation by [2+2+2] Cycloaddition Giving Rise to Sixfold Stereogenic C(sp^2^)–C(sp^3^) Atropisomers

In all examples of diastereodivergent catalysis presented so far, the diastereomeric symmetry arose from the presence of multiple stereogenic elements within one molecule. Beside these systems which follow the classical Le Bel–van ’t Hoff rule,^1,2^ in systems with higher-order stereogenicity, multiple stereoisomers—namely, enantiomers and diastereomers—arise from a single stereogenic unit. Pioneering work on rotamers with configurationally stable conformational states was conducted by the O̅ki group.^103^ Besides, examples such as fourfold stereogenicity in hexavalent stereocenters in octahedral metal complexes have been long known.^104^ However, their stereoselective preparation under catalyst control has not received much attention. Recently, catalyst stereocontrol over a molecule bearing a higher-order stereogenic element was reported for a sixfold stereogenic atropisomer. Based on the pioneering work of O̅ki on stable rotamers expanding the scope of C(sp^2^)–C(sp^2^) atropisomers, the isomers of product **77** with a stereogenic C(sp^2^)–C(sp^3^) single bond were investigated (Scheme 19).^105^ The rotational profile of this compound is characterized by six minima connected by sufficiently high rotational barriers to yield six configurationally stable rotamers, the configurations of which can be described using the Klyne–Prelog descriptors.^106^ The rigid ethenoanthracene fragment and the di-*ortho*-substituted aromatic part with a sterically highly demanding adamantyl group give rise to an interlocked system with high configurational stability. For stereoselective control over this scaffold, the Rh-catalyzed intramolecular [2+2+2] cycloisomerization of the three C≡C triple bonds of **94** showed to be effective, a strategy which was previously demonstrated to be successful in the construction of twofold stereogenic C(sp^2^)–C(sp^2^) axes.^107,108^ While spirocyclic bisphosphine ligand (*S*_a_)-**L17** rendered the direct single-step formation of the sixfold stereogenic axis highly enantio- and diastereoselective for the (+*ap*)-isomer (93:7:0:0:0:0, represented within a Newman plot in Scheme 19), NHC ligands were able to divert the stereoselectivity toward the *clinal* isomers (*−c*). The use of ligand (*R*)-**L18** in the Rh-catalyzed [2+2+2]-cycloisomerization of **94** thereby yielded (−*c*)-**95** in 78:5:12:5:0:0 stereoselectivity.

**Scheme 20:**
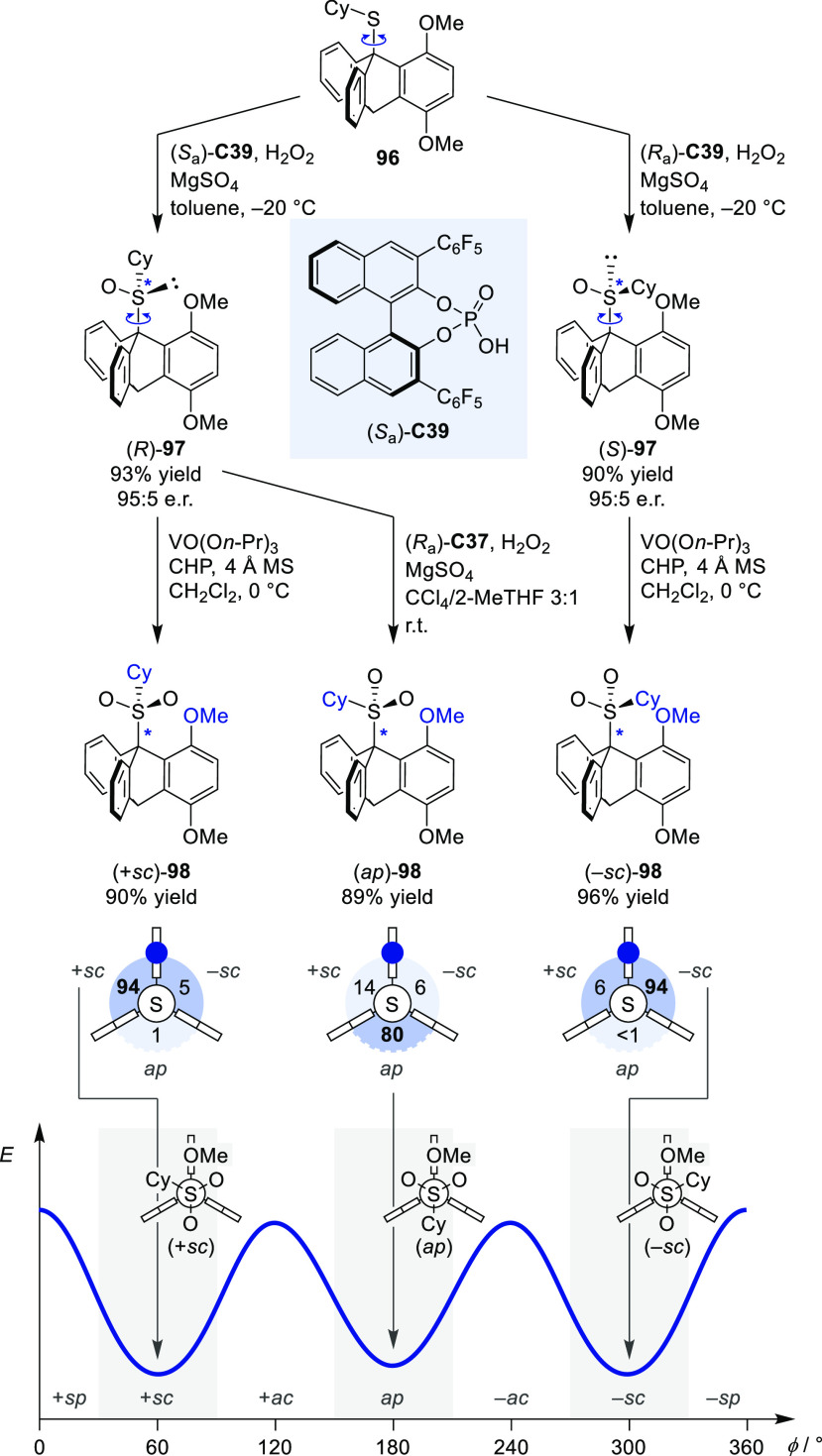
Catalyst-Controlled Access to All Three Stereoisomers of Atropisomeric Sulfone 98 with a Stereogenic C–S Axis CHP = cumene hydroperoxide.

The approach taken for the stereoselective preparation of **95** corresponds to the single-catalyst approach, as both enantioselectivity and diastereoselectivity are controlled by one catalyst in a single reaction. Related to sequential and iterative stereodivergent catalysis, atropisomeric sulfone **98** with a threefold stereogenic C–S axis was prepared in a two-step approach (Scheme 20).^109^ Enantioselective oxidation^110,111^ of rotationally dynamic thioether **96** with H_2_O_2_ under control of chiral phosphoric acid catalyst **C39** yielded (*R*)- and (*S*)-**97** respectively. Oxidation of the enantioenriched sulfoxide with a stereodynamic C–S axis yielded–depending on the catalyst and the reaction conditions–either the (*sc*)- or (*ap*)-diastereomer. With an enantiospecific transfer of the enantioenrichment of the sulfoxide stereocenter to the stereogenic axis, the (+*sc*)- and (−*sc*)-atropisomers could be accessed from the (*R*)- and (*S*)-sulfoxides respectively (up to 94:6:<1 (−*sc*):(+*sc*):(*ap*)). The symmetric (*ap*)-isomer was obtained in 80:20 d.r. from (*R*)-**97** using H_2_O_2_/(*R*_a_)-**C39** in a solvent mixture. A similar concept, where the configuration was addressed in two separate steps, was previously employed in the stereodivergent synthesis of fourfold stereogenic overcrowded alkenes, however exploiting reagent-controlled diastereodivergence.^112^

## 7 Catalyst Control over Alkene Configuration

**Scheme 21:**
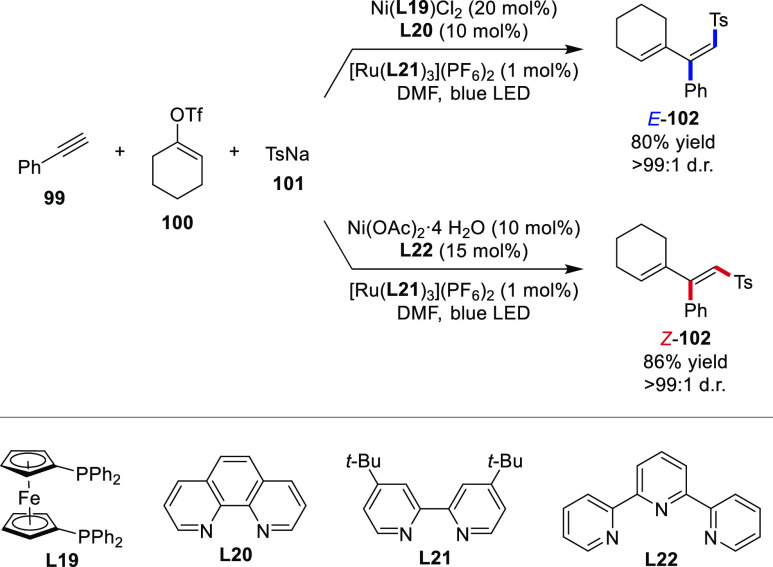
Direct Catalyst Control of the Double Bond Configuration in 1,3-Dienes

Alkenes are important structural motifs in many products and serve as platform intermediates for a variety of reactions. The selective synthesis of *E*- and *Z*-alkenes is thus highly desirable. While many of the reagent-based methodologies allow for selective access of one diastereomer by tuning reaction conditions and reagents, an entry to both alkene isomers from the same starting materials under direct catalyst control may substantially increase efficiency for streamlined access to geometrically well-defined building blocks. Yet, strategies for the diastereodivergent formation of alkenes are scarce. Catalytic methodologies for the double bond isomerization to the thermodynamically more stable configuration are well developed and photocatalysis elegantly established as suitable tool for the isomerization to the thermodynamically less stable isomer.^113^ While these alkene stereoisomerizations have been coupled with catalytic olefination reactions to selectively yield both diastereomers from the same starting materials,^114,115^ methods in which both alkene configurations are the direct result of distinct reaction pathways with the two catalysts are rare. For instance, a change of the catalyst from a Cu- to Au-based system was required to alter the reaction outcome of a double migratory cascade giving rise to *E*- and *Z*-1,3-dienes respectively^116^ while a very recent example employing photoredox and nickel dual catalysis achieved diastereodivergence under ligand control.^117^ Harnessing a synergistic effect between two ligands, the dual ligand system consisting of bisphosphine **L19** and 1,10-phenanthroline **L20** facilitated the three-component coupling between terminal alkyne **99**, vinyl triflate **100** and sodium sulfinate **101** to yield the *E*-alkene **102** with remarkable *E*/*Z*-selectivity (>99:1 d.r.) (Scheme 21). The *Z*-selective pathway was made available by tridentate terpyridine **L22** with a similar diastereoselectivity. Notably, an *E*/*Z*-isomerization succeeding the three-component coupling was precluded as the origin of diastereodivergence, and the direct catalyst control was attributed to differing mechanistic pathways. In the presence of bisphosphine ligand **L19**, the catalytic cycle is initiated by the oxidative addition of the vinyl triflate to Ni^0^(**L19**). After ligand exchange with **L20**, the resulting Ni^II^-complex is trapped by the vinyl radical independently formed from **99** and **101**. Reductive elimination selectively furnishes *E*-alkene **102**, and Ni^0^(**L19**) is regenerated by ligand exchange followed by reduction by the Ru-photocatalyst. In contrast to this mechanism, the *Z*-selective catalytic cycle proceeds without the involvement of Ni^0^ intermediates, and an alkenyl-Ni^I^-species, which can undergo *E*/*Z*-isomerization, is formed from **99** and **101** prior to the oxidative addition of **100**.

Compared with catalyst-controlled diastereodivergence in the synthesis of multiple stereocenters or stereogenic axes, the examples for *E*/*Z*-selective catalysis illustrate that achieving diastereodivergence in olefination reactions thus far requires drastic changes in the catalyst system and the mechanistic pathway. Catalytic methods granting divergent access to double bond diastereomers by simple variations of the ligand would thus significantly advance the methodologies.

## 8 Conclusion and Outlook

The field of diastereodivergent catalysis experienced a major leap over the past few years, which was also fueled by the development of new sophisticated catalytic concepts. The methodologies enable control not only over the configuration of stereocenters but also of atropisomers, higher-order stereogenic elements, and alkenes. While repeating fragments can be formed in iterative processes, single and dual catalyst methods evolved as a means to simultaneously set the configuration of two stereogenic elements within a single reaction. Thereby, transition metal complexes, organocatalysts, or combinations thereof are employed.^17^ However, due to the high requirements for catalyst, reagent, and substrate compatibility, diastereodivergent dual catalysis is still in its infancy, while it would allow facile access to all conceivable stereoisomers. The importance of diastereodivergent catalysis for the synthesis of compounds with medium to high stereochemical complexity can hardly be overestimated. Compared to enantiomers, which can be obtained by separating racemates, diastereodivergent catalysis allows synthetic access to stereochemical space which is not accessible otherwise in the frequent situation of a strong substrate bias. Conclusively, it is of great value to assess to what extent the methods developed for enantioselective catalysis are suitable to invert diastereoselectivity in the catalyst–substrate mismatched case, particularly when applied to chiral substrates that affect a medium to strong bias. The same relates to the question if methods that currently address one diastereomer can be further advanced to achieve selectivity for the elusive diastereomers. Moreover, methods that stereodivergently address more than two stereogenic elements with catalyst control would greatly advance the realm of each of the discussed concepts.
